# Supplementary material for: Multi-omics analysis defines highly refractory RAS burdened immature subgroup of infant acute lymphoblastic leukemia
Source: Nat Commun. 2022 Aug 30;13:4501. doi: 10.1038/s41467-022-32266-4 (PMC9427775; doi:10.1038/s41467-022-32266-4)
Supplement: Supplementary file 1 — Supplementary Information [file 41467_2022_32266_MOESM1_ESM.pdf]

Supplementary Information

Multi-omics analysis defines highly refractory RAS burdened immature subgroup of infant acute lymphoblastic leukemia

Tomoya Isobe[1,2,3,33], Masatoshi Takagi[4,33,*], Aiko Sato-Otsubo[1,33], Akira Nishimura[4], Genta Nagae[5], Chika Yamagishi[4], Moe Tamura[2], Yosuke Tanaka[6], Shuhei Asada[6,7], Reina Takeda[6], Akiho Tsuchiya[6], Xiaonan Wang[3], Kenichi Yoshida[8], Yasuhito Nannya[8], Hiroo Ueno[8,9], Ryo Akazawa[9], Itaru Kato[9], Takashi Mikami[9], Kentaro Watanabe[1], Masahiro Sekiguchi[1], Masafumi Seki[1], Shunsuke Kimura[1,10], Mitsuteru Hiwatari[1,11], Motohiro Kato[1], Shiro Fukuda[5], Kenji Tatsuno[5], Shuichi Tsutsumi[5], Akinori Kanai[12], Toshiya Inaba[13], Yusuke Shiozawa[8], Yuichi Shiraishi[14], Kenichi Chiba[14], Hiroko Tanaka[15], Rishi S Kotecha[16,17,18], Mark N Cruickshank[19], Fumihiko Ishikawa[20], Tomohiro Morio[4], Mariko Eguchi[21], Takao Deguchi[22,23], Nobutaka Kiyokawa[24], Yuki Arakawa[25], Katsuyoshi Koh[25], Yuki Aoki[26], Takashi Ishihara[27], Daisuke Tomizawa[28], Takako Miyamura[29], Eiichi Ishii[21], Shuki Mizutani[4], Nicola K Wilson[3], Berthold Göttgens[3], Satoru Miyano[15], Toshio Kitamura[6], Susumu Goyama[2], Akihiko Yokoyama[30], Hiroyuki Aburatani[5], Seishi Ogawa[8,31,32], Junko Takita[1,9,*]

<u>Inventory of Supplementary Information</u>

Supplementary Figure 1: Expression-based clustering validated IRX and HOXA subtypes of *KMT2A*-r infant ALL.

Supplementary Figure 2: Correlation between the *KMT2A* gene breakpoint and the IRX/HOXA axis.

Supplementary Figure 3: Methylation-based clustering of the 61 discovery cases of *KMT2A*-r infant ALL.

Supplementary Figure 4: Evaluation of the multi-omics clustering performance.

Supplementary Figure 5: Methylation-based classification of the extended cohort of *KMT2A*-r infant ALL.

Supplementary Figure 6: Global DNA methylation profiles and dual-omics markers of ICs.

Supplementary Figure 7: Differential expression of B cell genes in *KMT2A*-r infant ALL.

Supplementary Figure 8: Differential methylation of B cell genes in *KMT2A*-r infant ALL.

Supplementary Figure 9: Molecular profiles of *KMT2A*-r infant ALL compared to normal B cell progenitors.

Supplementary Figure 10: Differential signature activities between the ICs.

Supplementary Figure 11: Quality metrics and sensitivities of WES and deep-seq.

Supplementary Figure 12: Simple positivity of RTK-RAS pathway mutations were not significantly associated with clinical outcomes.

Supplementary Figure 13: Depths and coverages of WES and targeted deep sequencing in the extended cohort.

Supplementary Figure 14: Expression-based classification of a published dataset of *KMT2A*-r infant B-ALL.

Supplementary Figure 15: Flow cytometry sorting of diagnostic samples of MLL_96_073 and MLL_96_015.

Supplementary Figure 16: Heterogeneous developmental status of leukemia cells in MLL_96_073.

Supplementary Figure 17: Single cell transcriptome analysis of MLL_96_015.

Supplementary Figure 18: Cell type-specific enhancers defined by differential ChIP signals.

Supplementary Figure 19: Flow cytometry sorting scheme of human CB HSPCs.

Supplementary Table 1: Comparison of clinical characteristics in the five ICs.

Supplementary Table 2: Positive regulons of *IRX1* and *HOXA9* inferred by RTN.

Supplementary Table 3: Copy number alterations in the discovery cohort of 61 infants identified by WES and/or targeted deep sequencing.

Supplementary Table 4: Mutational analysis of the paired diagnostic and relapse samples of UT_INF_001.

Supplementary Table 5: Copy number alterations identified by WES and/or targeted deep sequencing in 12 infants from the extended cohort.

Supplementary Figure 1

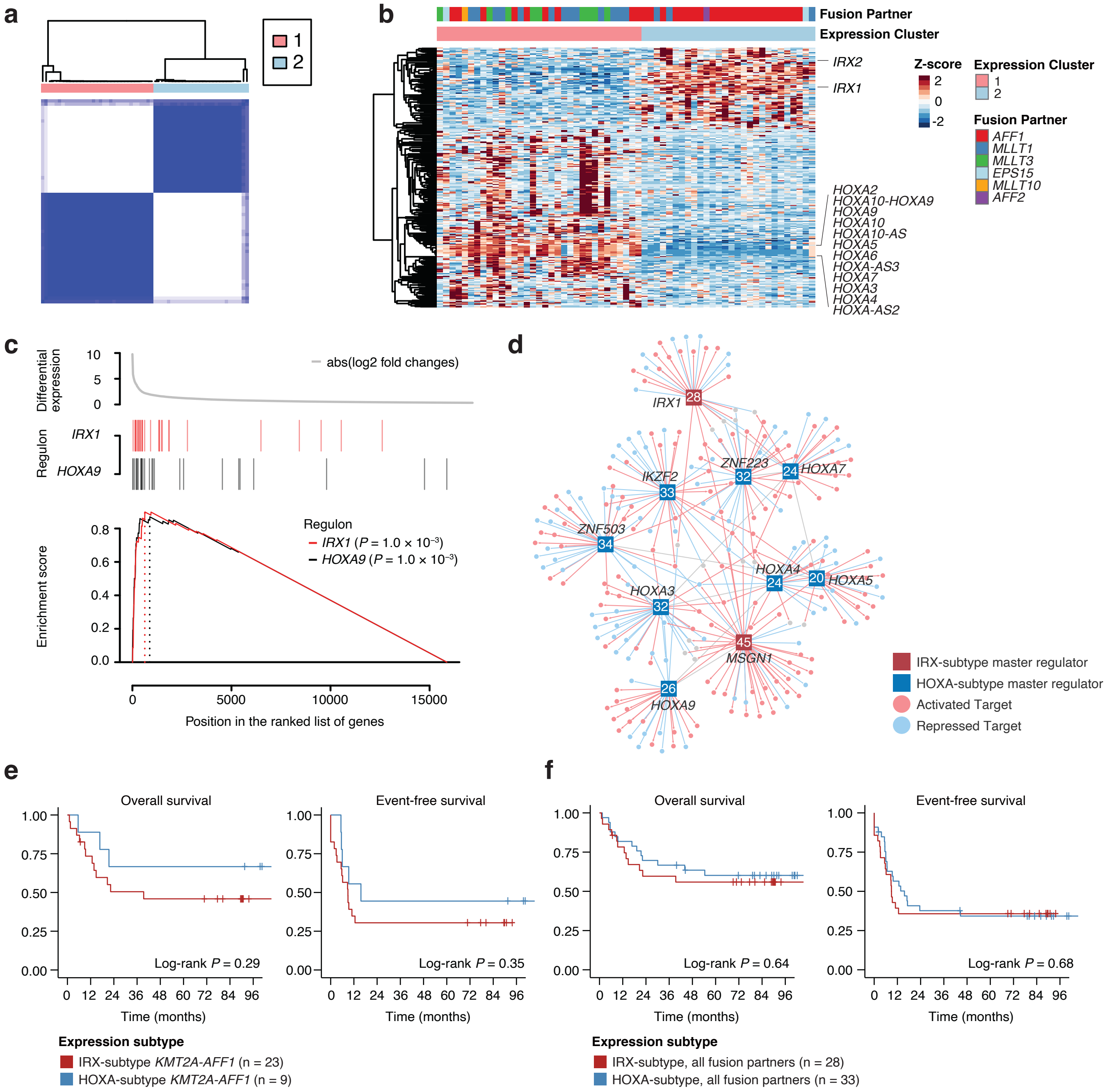

Supplementary Figure 1 I Expression-based clustering validated IRX and HOXA subtypes of *KMT2A*-r infant ALL.

a, Consensus matrix for k = 2. Consensus clustering was performed using 200 genes with the greatest median absolute deviation. **b**, Heatmap of the two clusters based on gene expression profiles. Significantly differentially expressed genes (BH-adjusted Wald test P<0.05 and log2 fold change >2) were used. Samples were arranged in the same order as in (a). **c**, Enrichment of target genes of master regulators (regulons) over differentially expressed genes between the two clusters. Enrichment plots were generated for the two most significant regulons (*IRX1* and *HOXA9*). Transcriptional networks were reconstructed using RTN (ref.[44]) through 1,000 permutations and master regulators were then inferred. P values are raw P values derived from one-tailed gene set enrichment analysis (GSEA-1T) implemented in RTN. **d**, Top 10 master regulators controlling differential expression between the two gene expression clusters of infant ALL. Rectangular nodes represent master regulators (red, upregulated in IRX-subtype; blue, upregulated in HOXA-subtype). Circular nodes represent positively (red) or negatively (blue) regulated target genes. The number of target genes is indicated on each master regulator node. **e**-**f**, Survival analysis of *KMT2A-AFF1* cases (e) and all cases (f) based on the IRX vs. HOXA expression subtypes.

Supplementary Figure 2

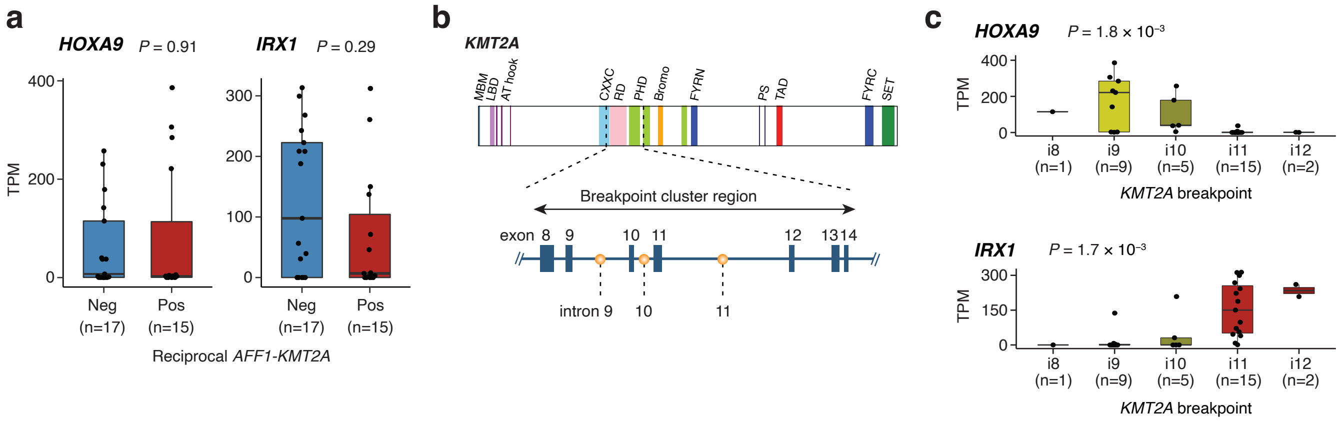

Supplementary Figure 2 | Correlation between the *KMT2A* gene breakpoint and the IRX/HOXA axis.

a, Expression levels of *HOXA9* and *IRX1* based on the positivity of the reciprocal *AFF1-KMT2A* fusion transcripts. Box plots show median and first/third quartiles. The whisker extends from the smallest to the largest values within 1.5 × IQR from the box hinges. *P* values are from two-sided Wilcoxon rank sum test. **b**, Schematic illustration of the structure and breakpoint cluster region of KMT2A. The three most common breakpoints (intron 9, 10 and 11) are indicated. MBM, MENIN binding motif; LBD, LEGDF binding domain; RD, repression domain; PS, processing site; TAD, transactivation domain. **c**, Expression levels of *HOXA9* and *IRX1* in *KMT2A-AFF1*-positive infants based on the *KMT2A* gene breakpoint. *P* values are from Kruskal-Wallis test. Box plots show median and first/third quartiles. The whisker extends from the smallest to the largest values within 1.5 × IQR from the box hinges.

Supplementary Figure 3

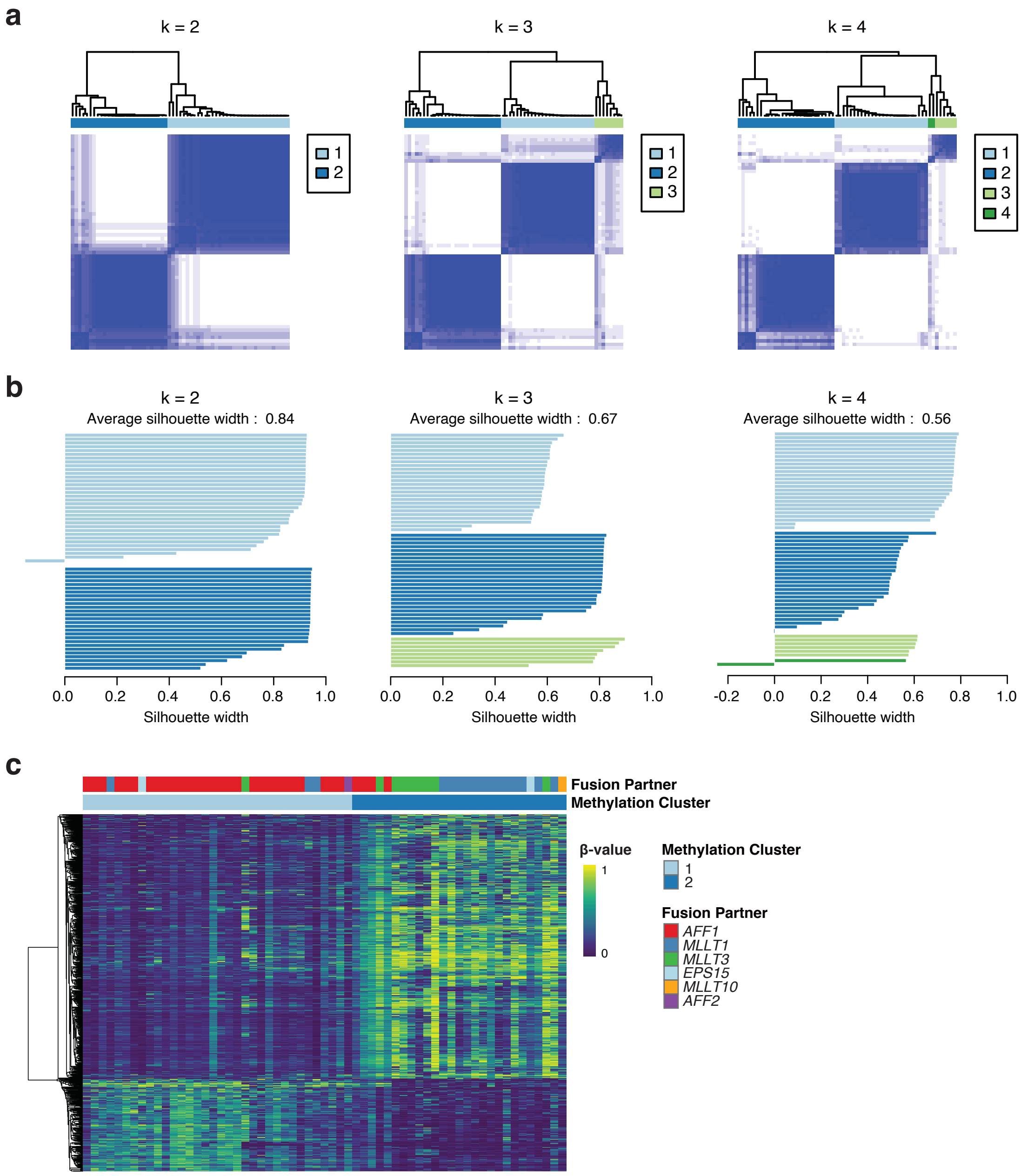

Supplementary Figure 3 I Methylation-based clustering of the 61 discovery cases of *KMT2A*-r infant ALL.

a, Consensus matrix for k = 2 to k = 4. Consensus clustering was performed using 1000 probes with the greatest median absolute deviation of M-values. **b**, Silhouette plots for k = 2 to k = 4. **c**, DNA methylation heatmap of the methylation-based clusters (k = 2). The top 1000 significantly differentially methylated probes between clusters were used.

Supplementary Figure 4

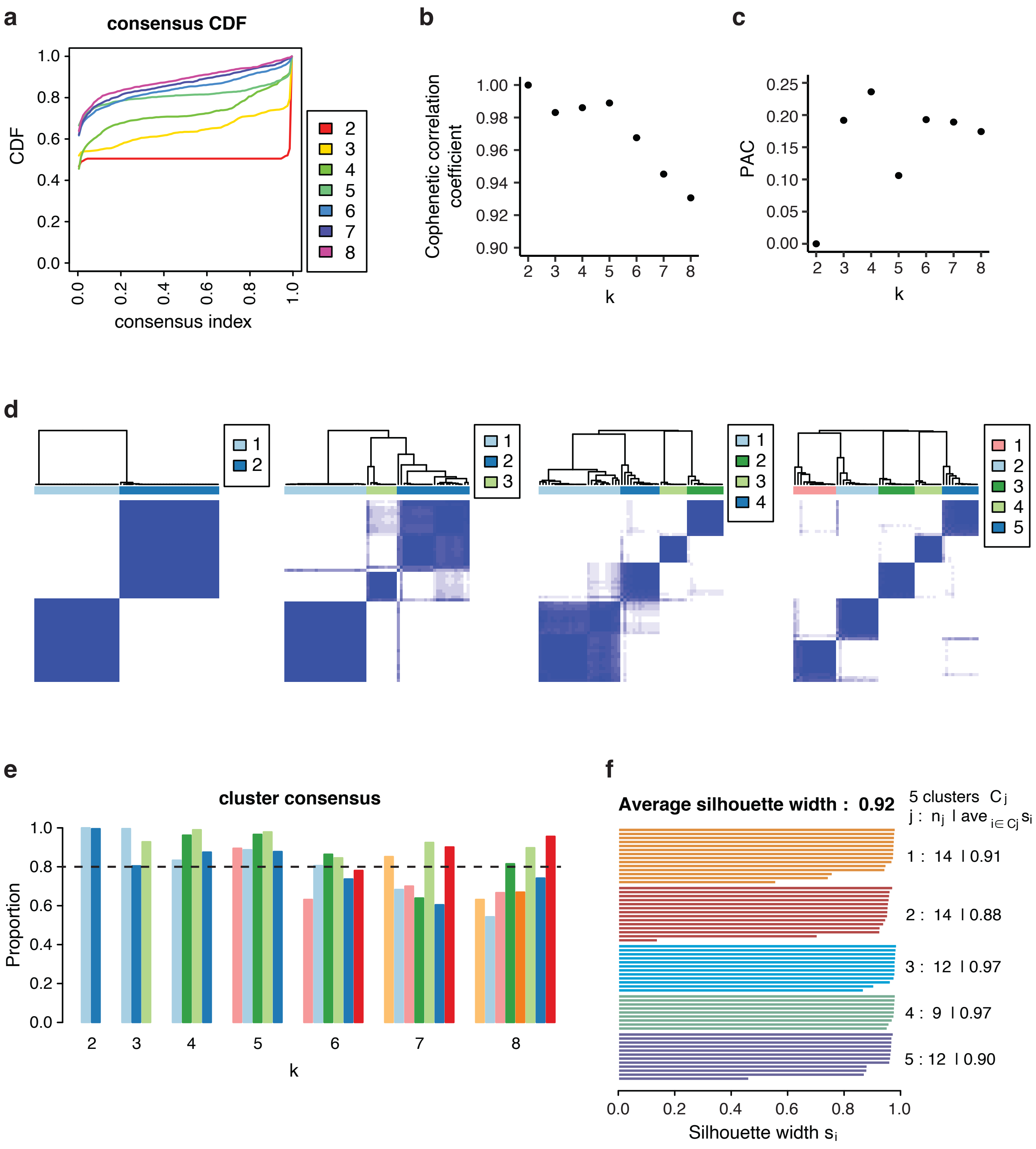

Supplementary Figure 4 | Evaluation of the multi-omics clustering performance.

a-**c**, Unsupervised consensus clustering on the dual-omics (transcriptome and methylome) molecular profiles integrated with the SNF algorithm. Cumulative distribution function (CDF) curves (a), cophenetic correlation coefficients (b), and proportion of ambiguous clustering (PAC) are plotted for k = 2 to k = 8. **d**, Consensus matrix heatmaps for k = 2 to k = 5. **e**, Cluster consensus plots for k = 2 to k = 8, showing the means of all pairwise consensus values between the members of each cluster. **f**, Silhouette plot for k = 5 clusters.

Supplementary Figure 5

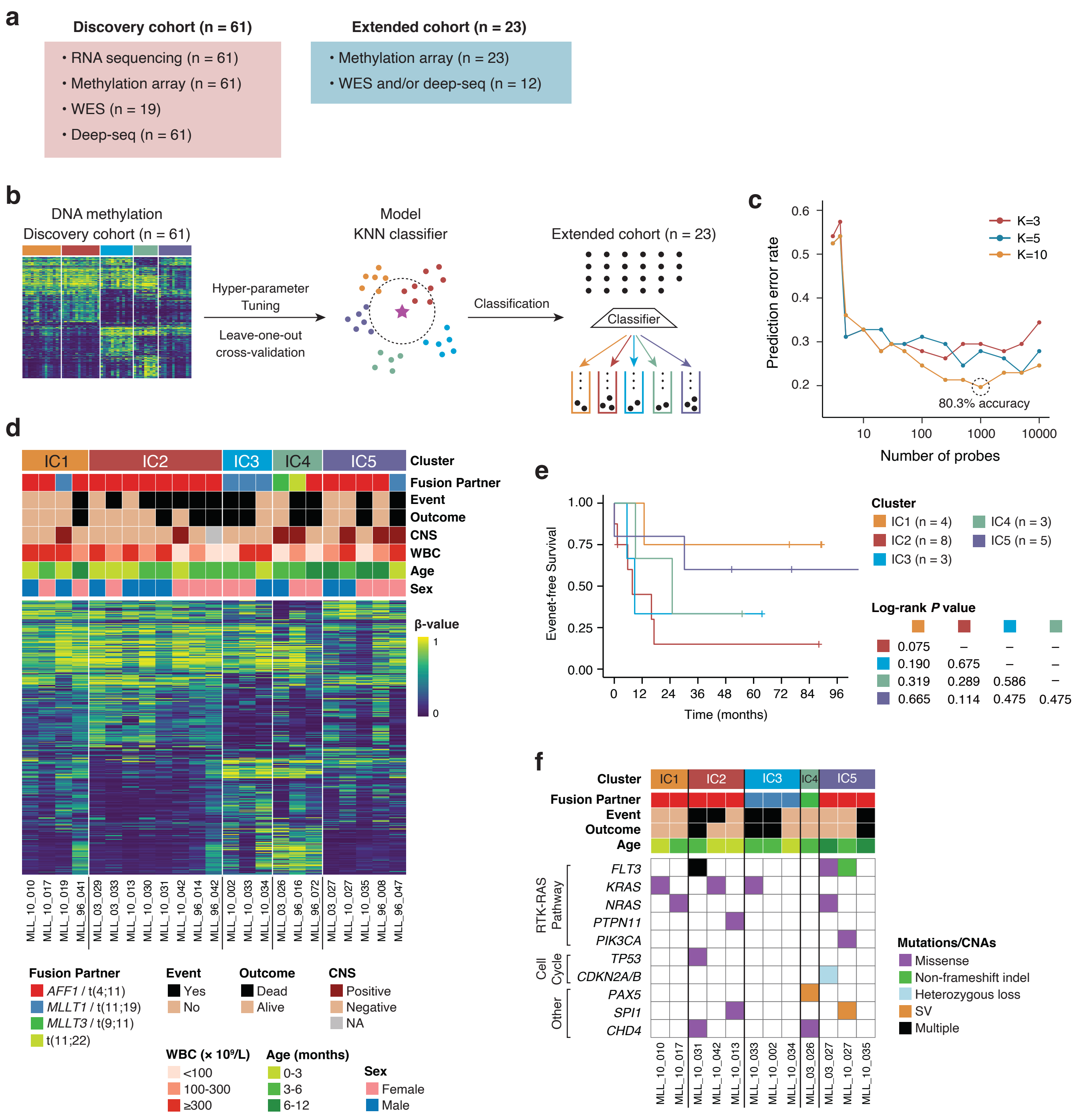

Supplementary Figure 5 I Methylation-based classification of the extended cohort of *KMT2A*-r infant ALL.

a, Summary of analyses conducted in the discovery (n = 61) and extended (n = 23) cohorts. WES, whole exome sequencing; deep-seq, targeted deep sequencing. **b**, Schematic illustration of the procedures for KNN model building and cluster assignment for the extended cases. **c**, Plot of the prediction error rates for the hyper-parameter pairs used in the leave-one-out cross-validation procedure. **d**, Methylation β-values of the extended cases (n = 23) are shown for the probes used in Fig. 1a. The veritical order of the methylation probes is also the same as in Fig. 1a. Clinicopathological features are annotated above the methylation heatmap. NA, not available. **e**, Survival analysis of the extended cases based on the assigned IC labels. **f**, Mutations, CNAs and SVs identified in the extended cases evaluated with WES and/or deep-seq (n = 12).

Supplementary Figure 6

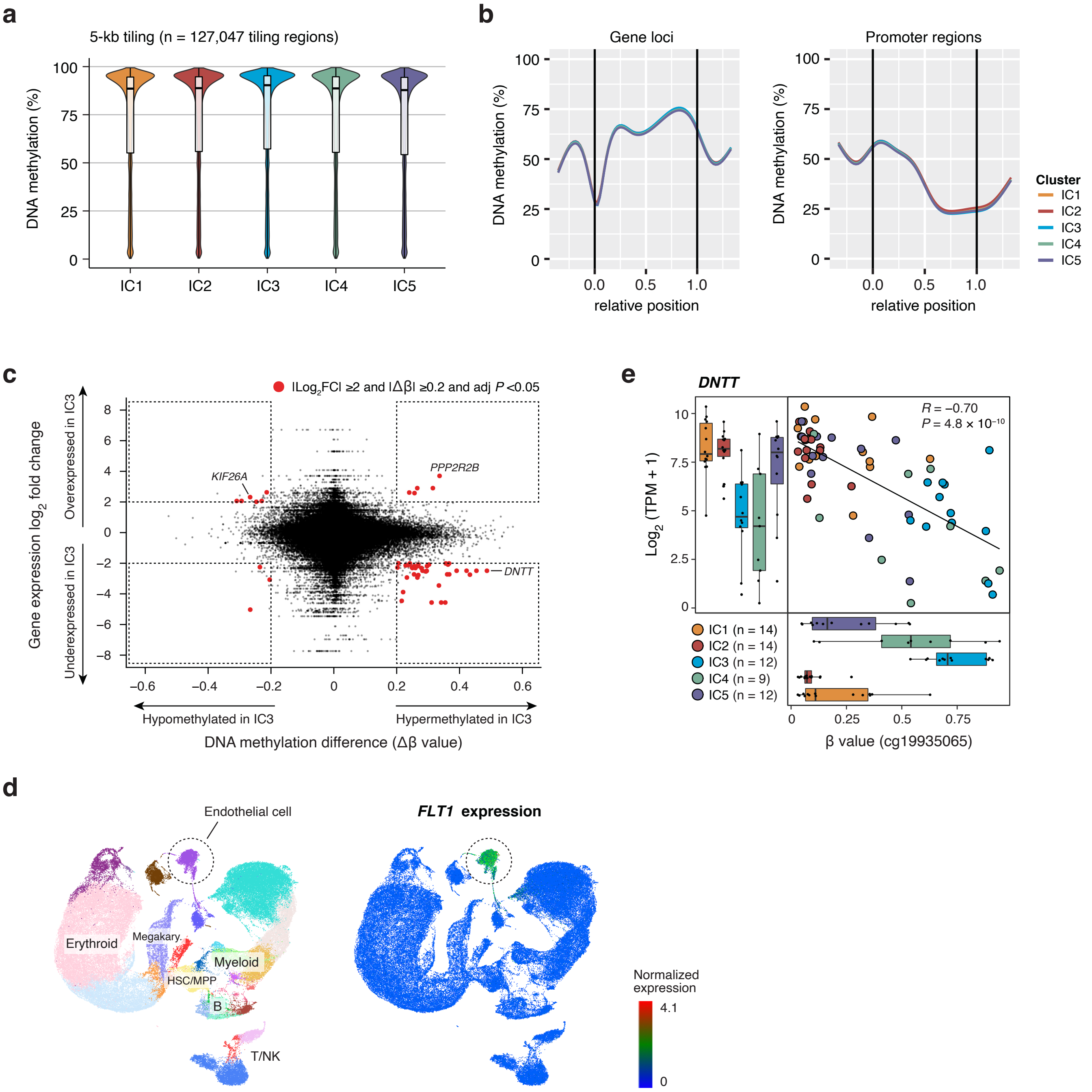

Supplementary Figure 6 | Global DNA methylation profiles and dual-omics markers of ICs.

a, Global DNA methylation levels of ICs. Probe-wise methylation levels were averaged for every 5-kilobase window, and the methylation levels of tiling regions are summarized in violoin plots. Box plots show median and first/third quartiles. **b**, Smoothed methylation levels across all gene (left) and promoter (right) loci. Methylation levels are summarized according to relative position within genes and promoters. **c**, Integrative scatter plots contrasting expression differences with DNA methylation differences in IC3. Genes and probes with significant differences in gene expression (|$\log_2$ fold change| ≥2 and two-sided adjusted P <0.05) and DNA methylation (|Δβ| ≥0.2 and two-sided adjusted P <0.05) are highlighted in red. **d**, Expression of *FLT1* in the fetal liver hematopoiesis atlas (ref.[22]). Major cell types (left) and cell-wise expression (right) are shown in the UMAP representation. **e**, Correlated gene expression and DNA methylation of *DNTT*. Pearson correlation coefficient (R) and raw Pearson correlation P-value are indicated. Box plots show median and first/third quartiles.

Supplementary Figure 7

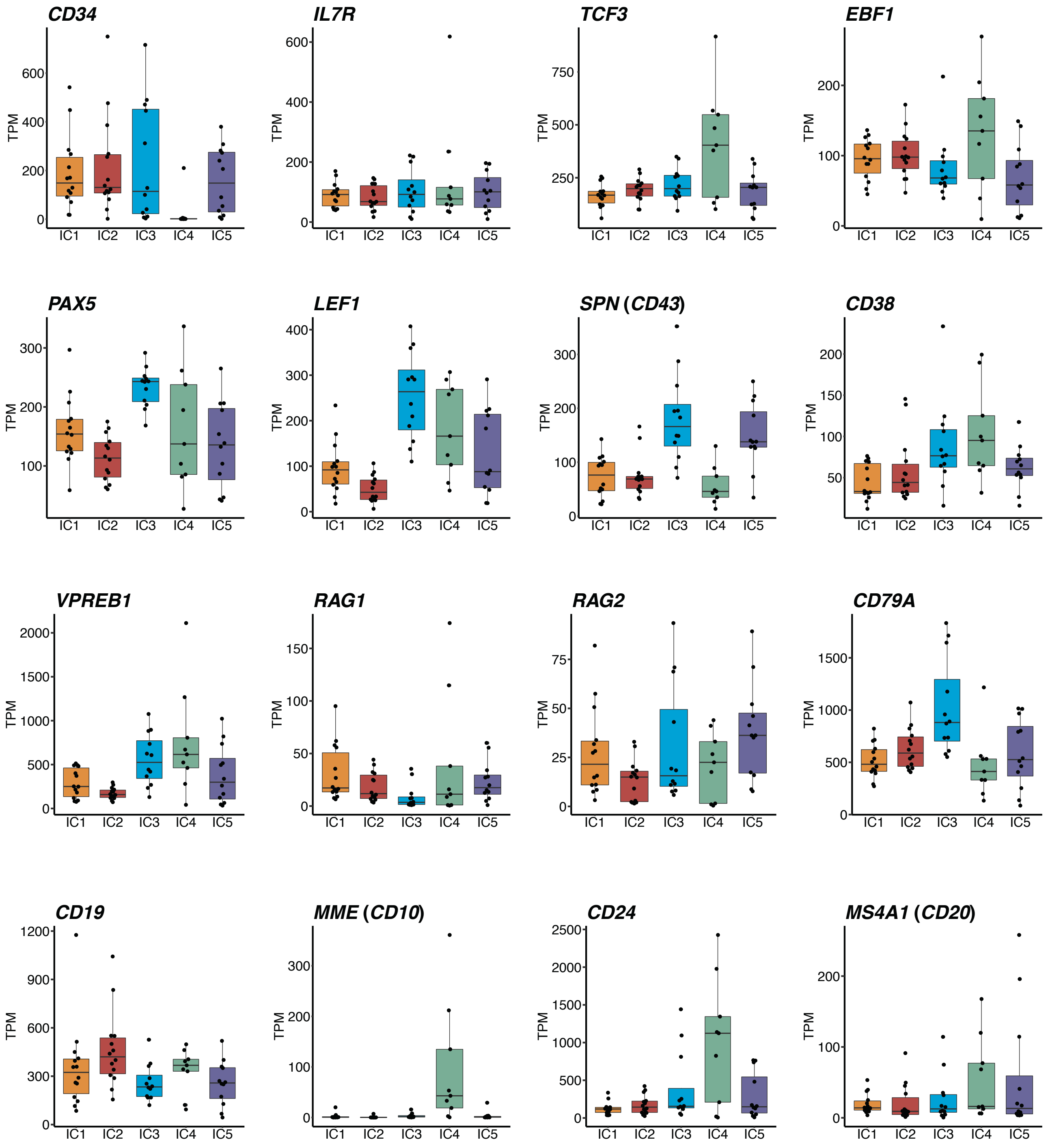

Supplementary Figure 7 I Differential expression of B cell genes in *KMT2A*-r infant ALL.

Expression levels (TPM) of genes associated with B cell development. All 61 cases of the discovery cohort are included: IC1 (n=14), IC2 (n=14), IC3 (n=12), IC4 (n=9) and IC5 (n=12). Box plots show median and first/third quartiles. The whisker extends from the smallest to the largest values within 1.5 × IQR from the box hinges.

Supplementary Figure 8

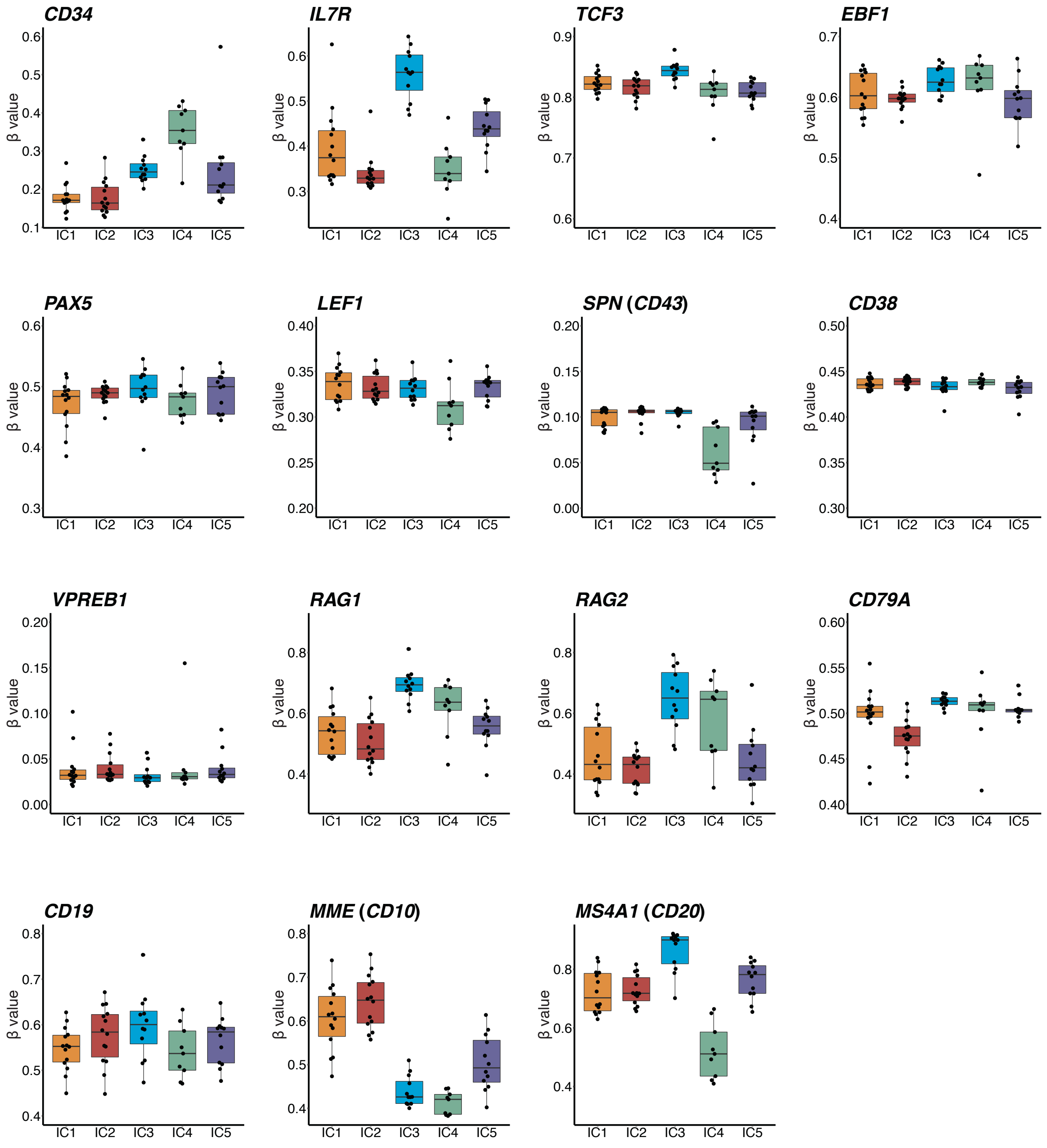

Supplementary Figure 8 | Differential methylation of B cell genes in *KMT2A-r* infant ALL.

Methylation β values of genes associated with B cell development. The same genes are shown as in Supplementary Figure 7, with the exception of *CD24*, for which no evaluable methylation probes were available. Gene-level β values were calculated as the mean of probe-level β values within each gene locus. All 61 cases of the discovery cohort are included: IC1 (n=14), IC2 (n=14), IC3 (n=12), IC4 (n=9) and IC5 (n=12). Box plots show median and first/third quartiles. The whisker extends from the smallest to the largest values within 1.5 × IQR from the box hinges.

Supplementary Figure 9

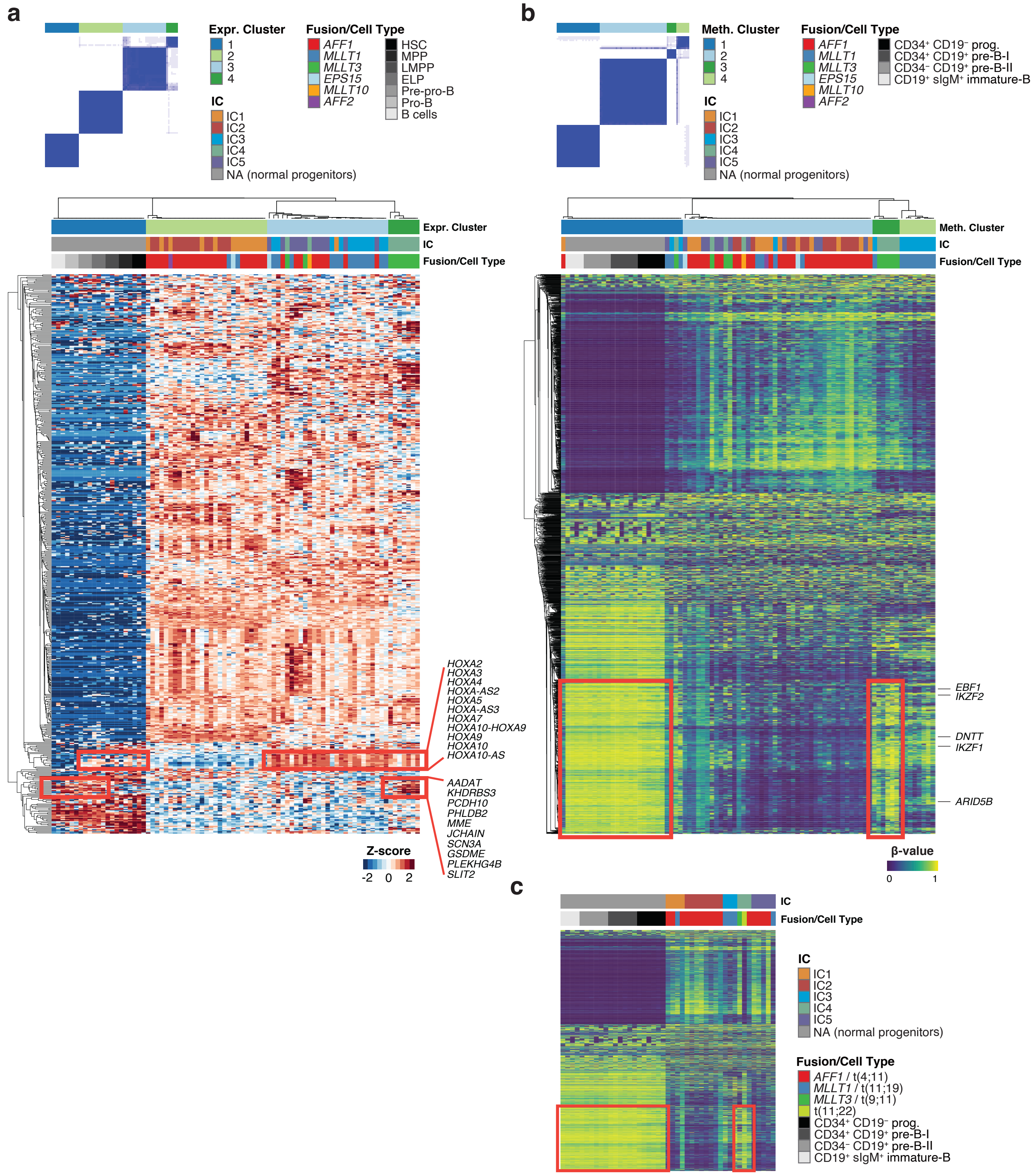

Supplementary Figure 9 | Molecular profiles of *KMT2A*-r infant ALL compared to normal B cell progenitors.

a-b, Expression-based (a) and Methylation-based (b) consensus clustering of infant ALL (n = 61) and normal B cell progenitors (n = 21 for expression[24]; n = 22 for methylation[25]). Consensus matrix plots for k = 4 (upper left panels) and heatmaps (lower panels) are shown. Consensus clustering was performed using 500 genes (a) and 2000 probes (b) with the greatest median absolute deviation. Characteristic expression and methylation signatures shared by subsets of normal progenitors and infant ALL are highlighted by red rectangles. **c**, Methylation heatmap of the extended cases (n = 23) for the same probes as in (b).

Supplementary Figure 10

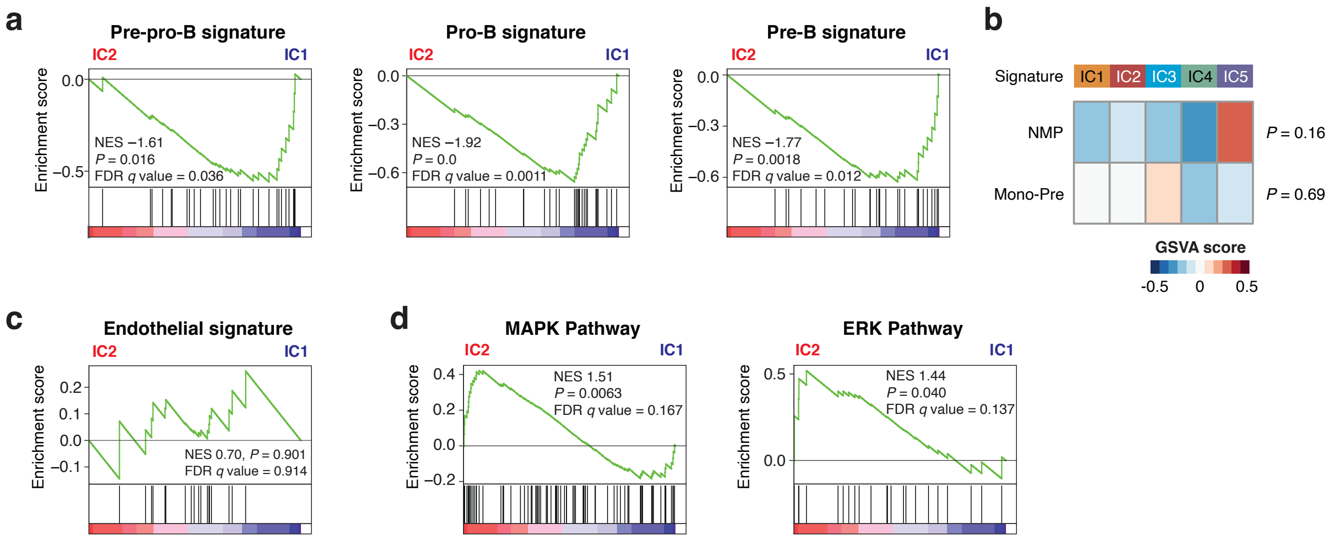

Supplementary Figure 10 I Differential signature activities between the ICs.

a, Enrichment analysis of B cell developmental signatures comparing IC2 and IC1. *P* values are raw *P* values from GSEA. False discovery rates (FDRs) adjusted for multiple testing are also shown. NES, normalized enrichment score. **b**, GSVA enrichment analysis of myeloid lineage signatures between the five ICs. Median value for each signature in each cluster is plotted. Kruskal-Wallis *P* values are indicated. NMP, neutrophil-myeloid progenitor; Mono-Pre, Monocyte precursor. **c-d**, Enrichment analysis of endothelial cell signature (c) and RAS downstream MAPK and ERK pathways (Biocarta; d) comparing IC2 against IC1. *P* values are raw *P* values from GSEA. FDRs adjusted for multiple testing are also shown.

Supplementary Figure 11

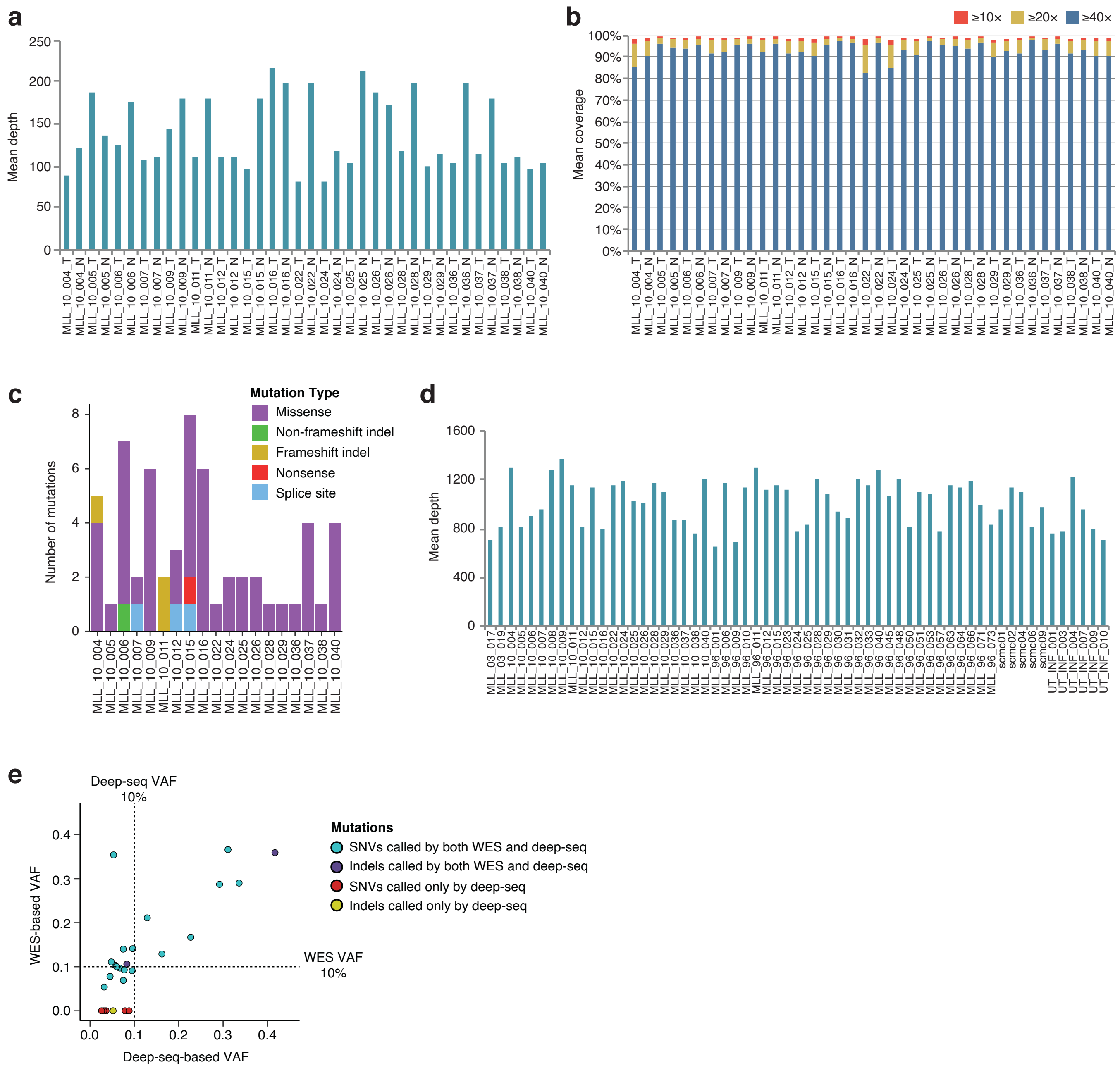

Supplementary Figure 11 | Quality metrics and sensitivities of WES and deep-seq.

a, Depths of WES on tumor (T) and normal (N) DNA samples from 19 cases in the discovery cohort (mean depth = 140). **b**, Coverages of the exome sequences; 98% of exome sequences were analyzed at a depth greater than 20×. **c**, The number of somatic SNVs and indels identified by WES. **d**, Depths of targeted deep sequencing on tumor DNA samples from all 61 cases in the discovery cohort (mean depth = 998). **e**, VAFs of 26 mutations identified with WES and/or deep-seq in the 19 discovery cases for which both WES and deep-seq were performed. In the19 mutations with deep-seq VAFs <10%, six mutations were not detected with WES, and therefore depicted as having a WES VAF = 0.

Supplementary Figure 12

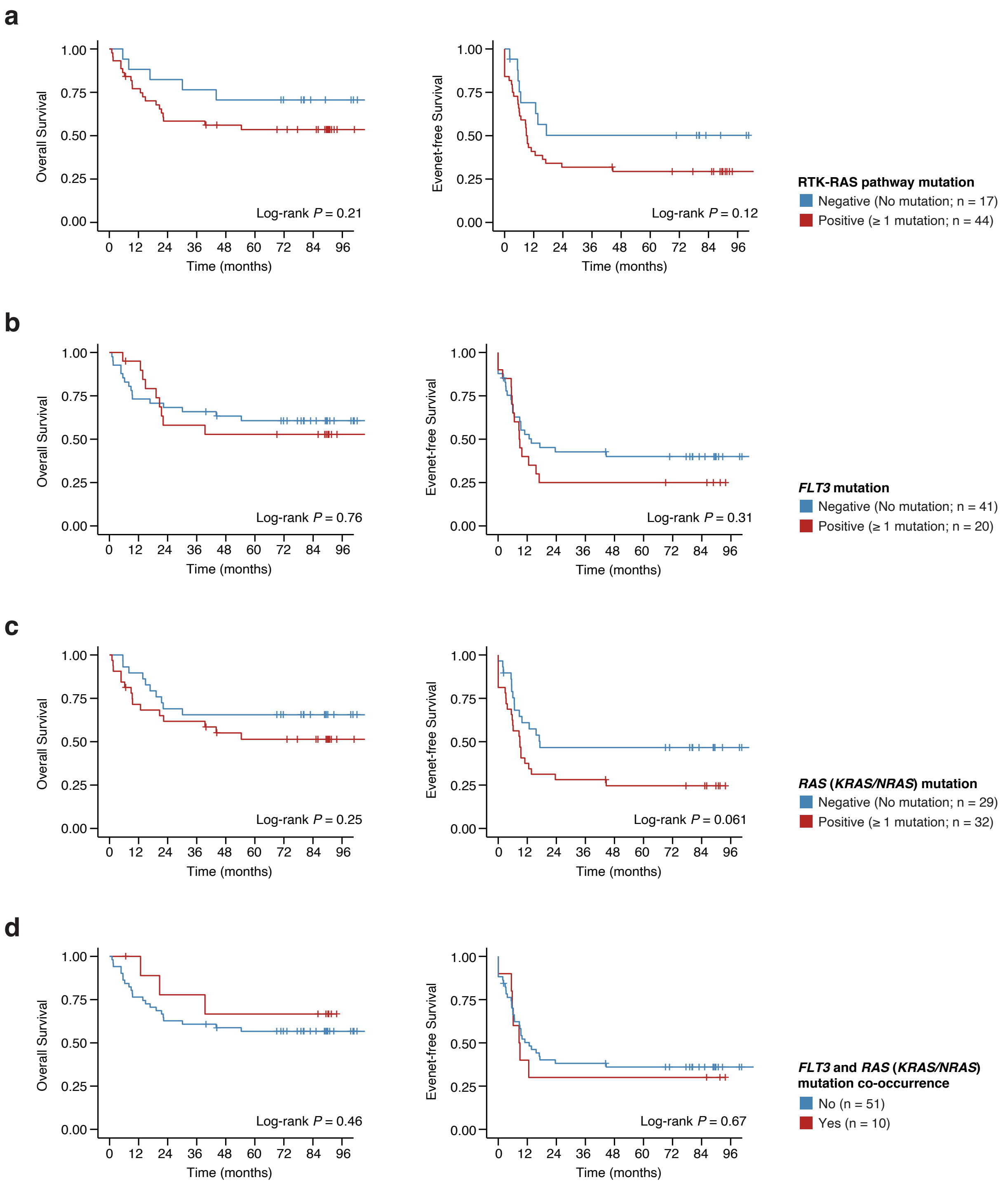

Supplementary Figure 12 | Simple positivity of RTK-RAS pathway mutations were not significantly associated with clinical outcomes. a-d, Survival analysis of *KMT2A*-r infant ALL based on the positivity of RTK-RAS pathway mutations (a), *FLT3* mutations (b), *RAS* (*KRAS* and/or *NRAS*) mutations (c), and co-occurrence of *FLT3* and *RAS* mutations.

Supplementary Figure 13

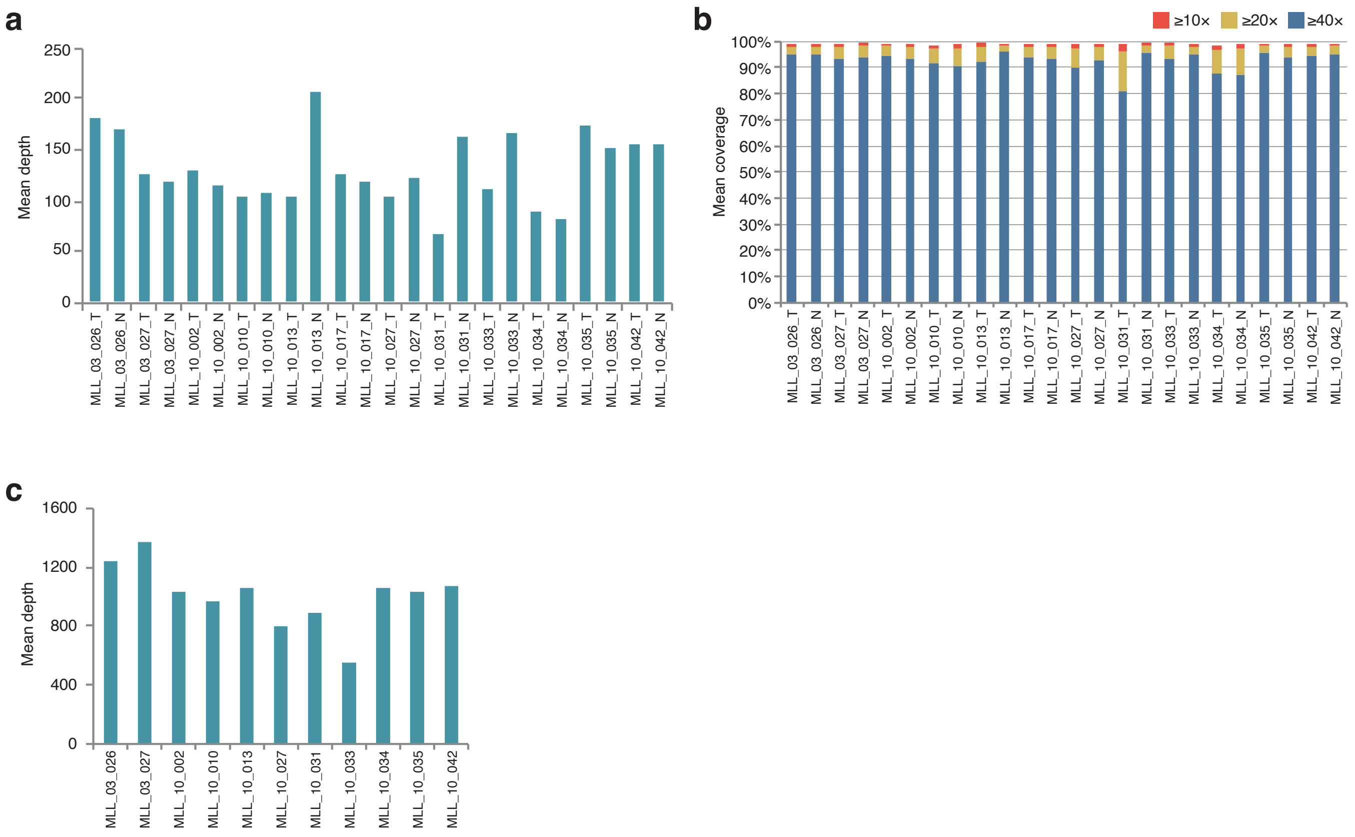

Supplementary Figure 13 I Depths and coverages of WES and targeted deep sequencing in the extended cohort.

a, Depths of WES on tumor (T) and normal (N) DNA samples from 12 cases in the extended cohort (mean depth = 131). **b**, Coverages of the exome sequences; 98% of exome sequences were analyzed at a depth greater than 20×. **c**, Depths of targeted deep sequencing on tumor DNA samples from 11 cases in the extended cohort (mean depth = 1005).

Supplementary Figure 14

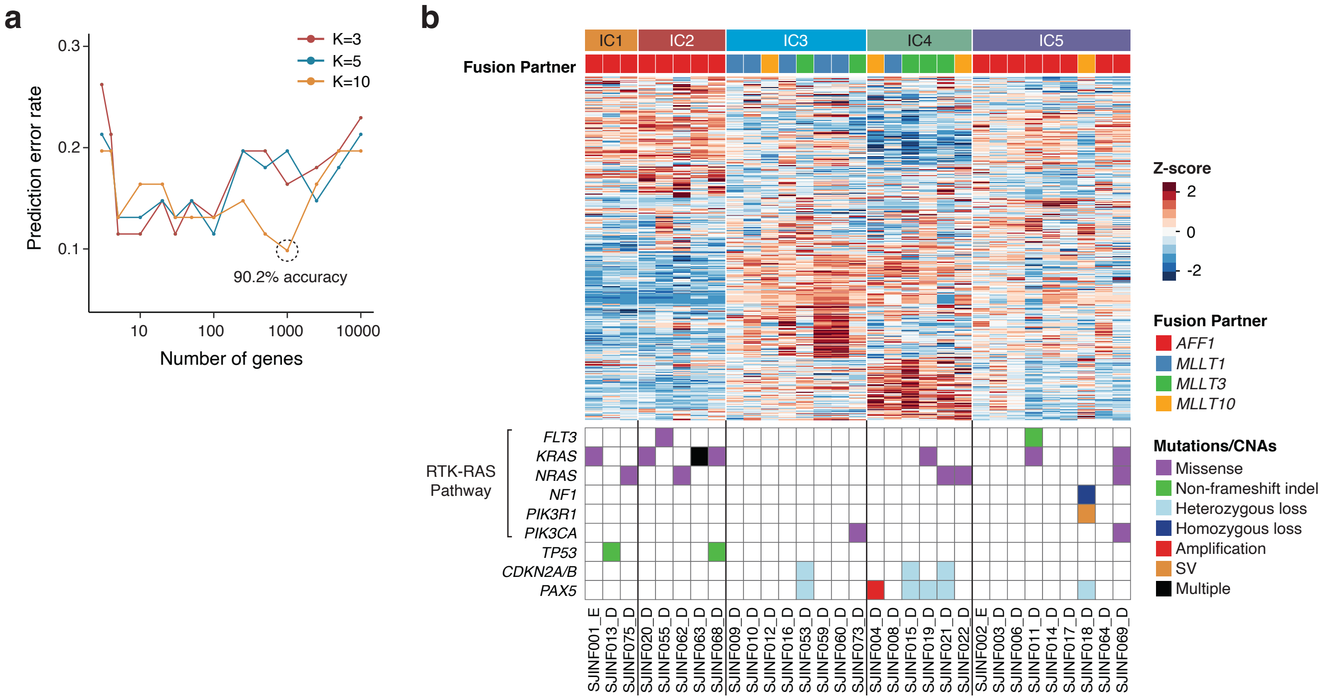

Supplementary Figure 14 | Expression-based classification of a published dataset of *KMT2A*-r infant B-ALL.

a, Prediction error rates of the expression-based KNN classifier generated with the 61 cases of our discovery cohort. The plotted hyper-parameter pairs were evaluated with leave-one-out cross-validation. **b**, Gene expression and mutation profiles of the St Jude cohort of infant B-ALL (EGAS00001000246; n = 31). The IC labels were predicted using the KNN model trained with our discovery cohort. The same expression heatmap genes are used in the same order as in Fig. 1a.

Supplementary Figure 15

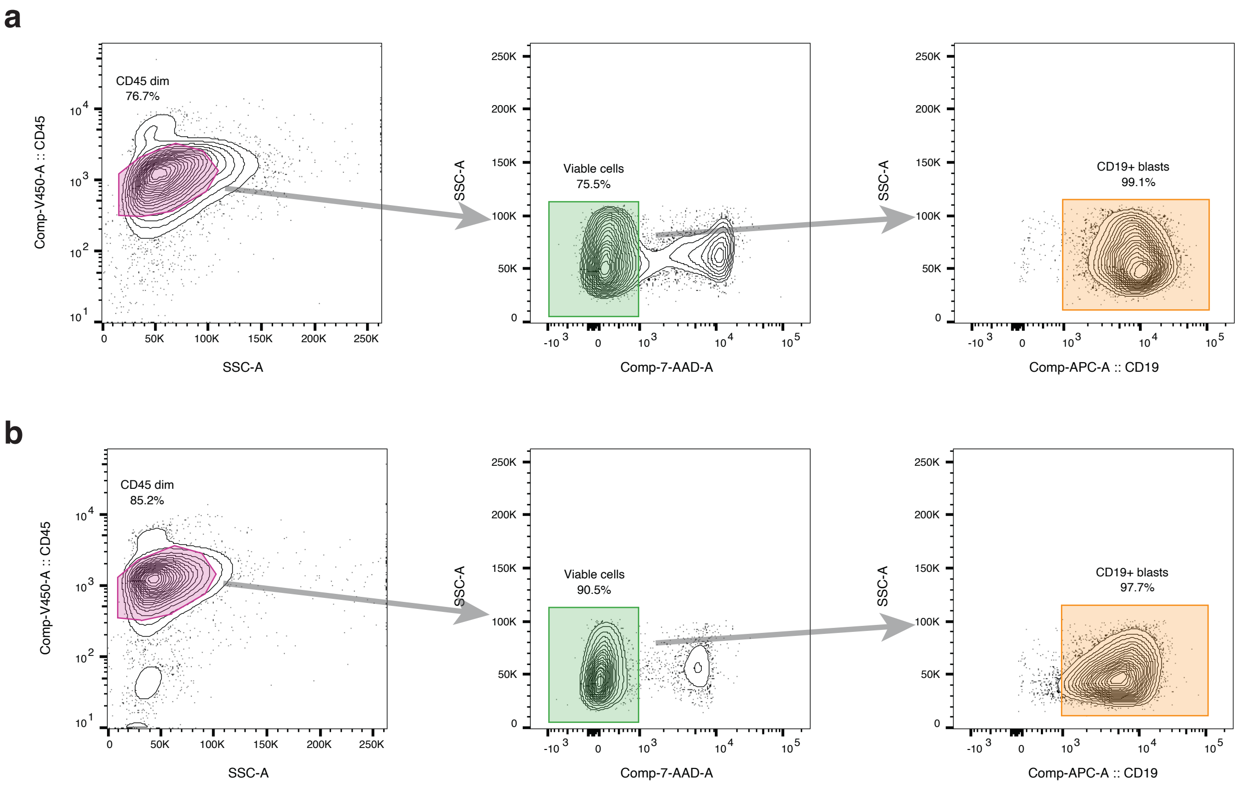

Supplementary Figure 15 I Flow cytometry sorting of diagnostic samples of MLL_96_073 and MLL_96_015.

a-b, The displayed gating strategy was used to enrich viable leukemic blasts ($CD45^{dim}$ $CD19^{+}$) from the diagnostic samples of MLL_96_073 (a) and MLL_96_015 (b). The sorted leukemia cells were then subjected to single-cell transcriptome library preparation.

Supplementary Figure 16

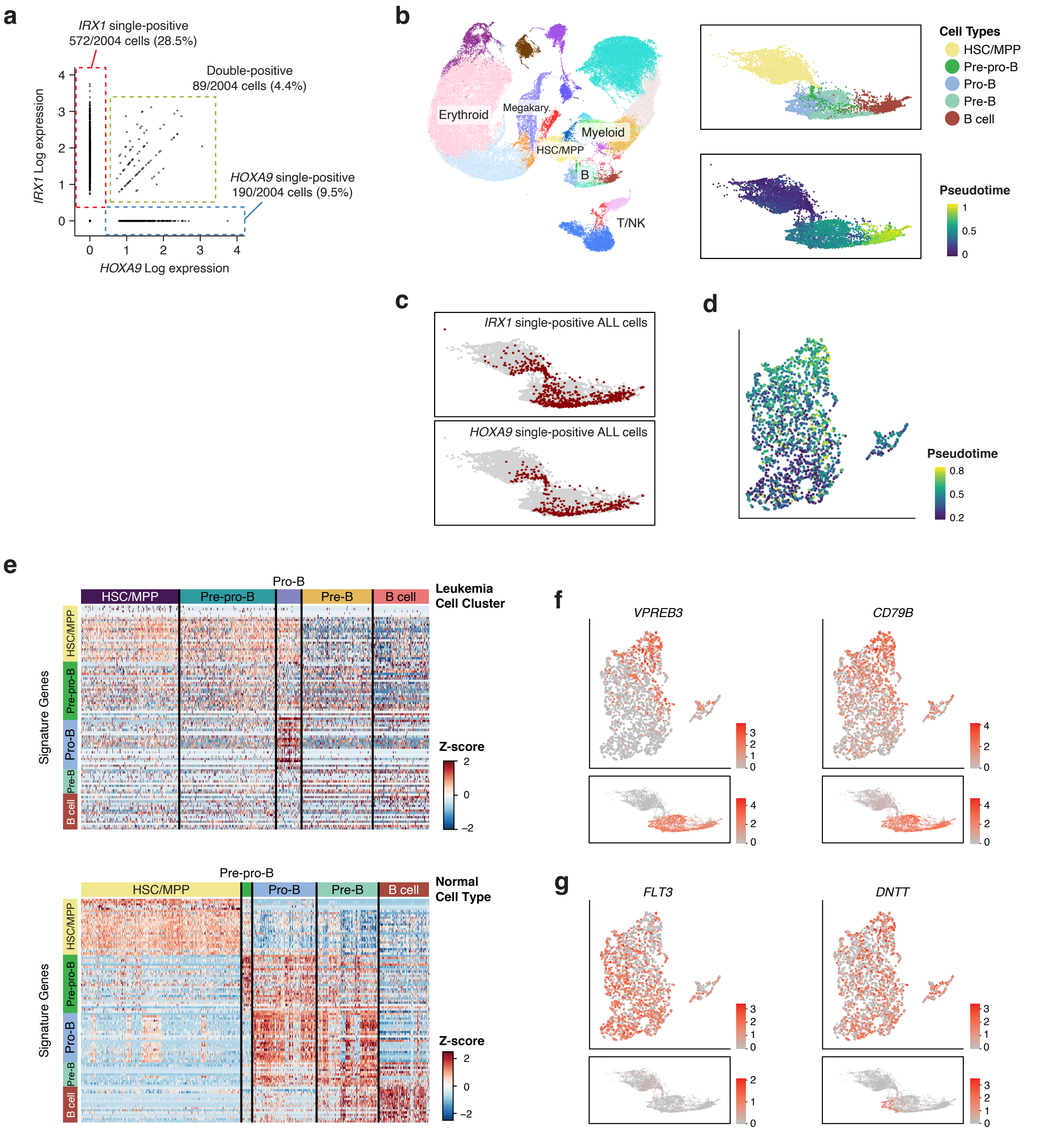

Supplementary Figure 16 | Heterogeneous developmental status of leukemia cells in MLL_96_073.

a, Log normalized expression of *IRX1* and *HOXA9* in the 2004 cells from MLL_96_073. **b**, Differentiation pseudotime of normal fetal liver B-cell progenitors. **c**, Nearest neighbors of *IRX1*-positive and *HOXA9*-positive leukemia cells mapped on the fetal liver atlas. **d**, Estimated B-cell differentiation pseudotime of leukemia cells in MLL_96_073. **e**, Expression heatmaps of B-cell stage markers in leukemia cells (upper panel) and normal fetal liver progenitors (lower panel). Genes are ordered as listed in Supplementary Data 6. **f-g**, Ordered (f) and aberrant (g) expression of B-cell developmental genes in leukemia cells (upper panels). Expression patterns in normal progenitors are shown in lower panels. Color scales indicate log normalized expression.

Supplementary Figure 17

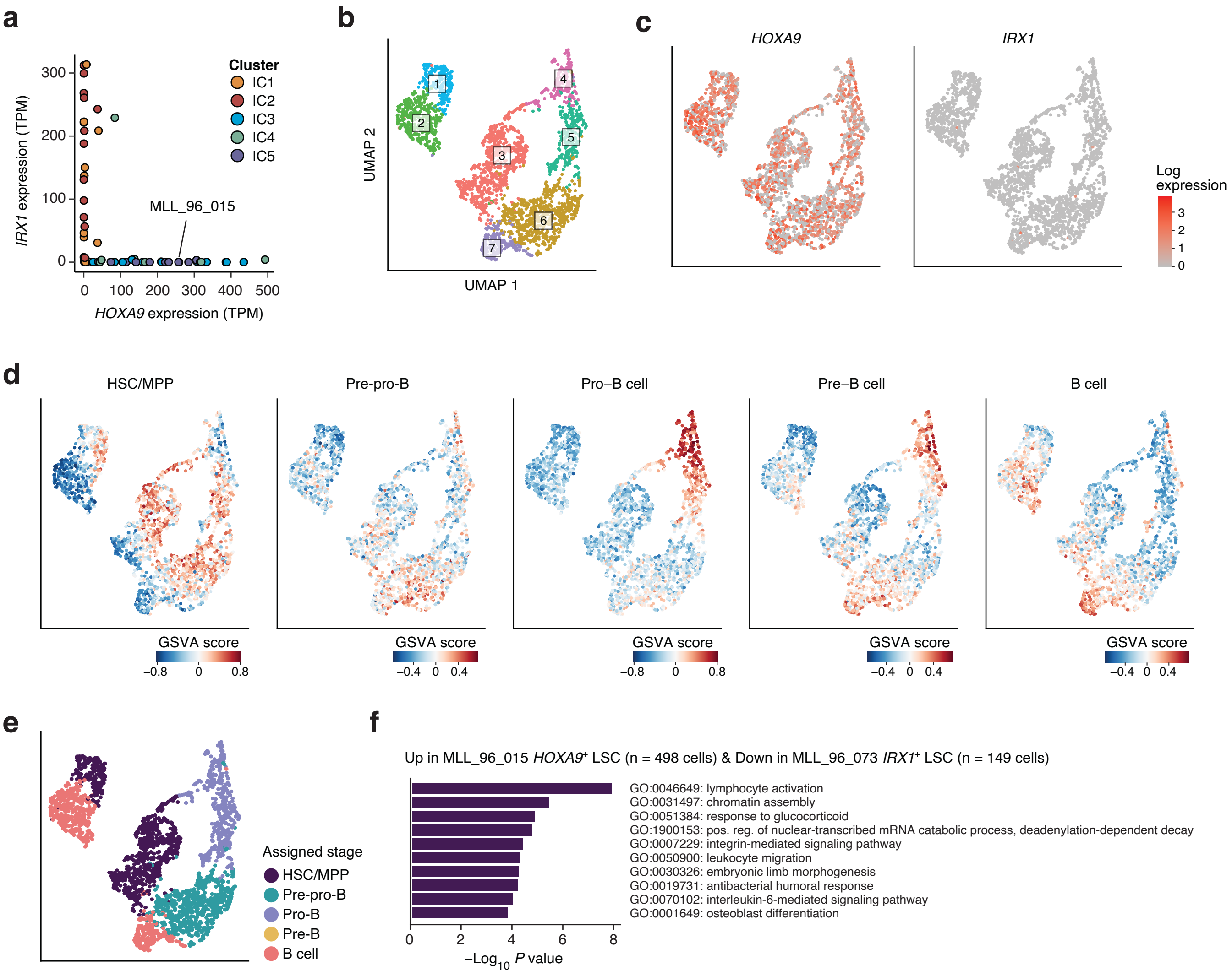

Supplementary Figure 17 | Single cell transcriptome analysis of MLL_96_015.

a, A *HOXA9* single-positive infant subjected to single-cell RNA sequencing (MLL_96_015) is indicated in a two-dimensional plot of *IRX1* and *HOXA9* gene expression in patient samples. **b**, UMAP visualization of the single-cell transcriptome of MLL_96_015. Clusters were identified with the Louvain method. **c**, Log normalized expression values of *IRX1* and *HOXA9* in the leukemia cells of MLL_96_015. **d**, Enrichment of B-lineage developmental signatures in the single-cell transcriptome of MLL_96_015. **e**, Assigned developmental stages of the leukemia cells from MLL_96_015. **f**, Enriched Gene Ontology terms comparing *HOXA9* single-positive LSCs from MLL_96_015 (n = 498 cells) vs. *IRX1* single-positive LSCs from MLL_96_073 (n = 149 cells).

Supplementary Figure 18

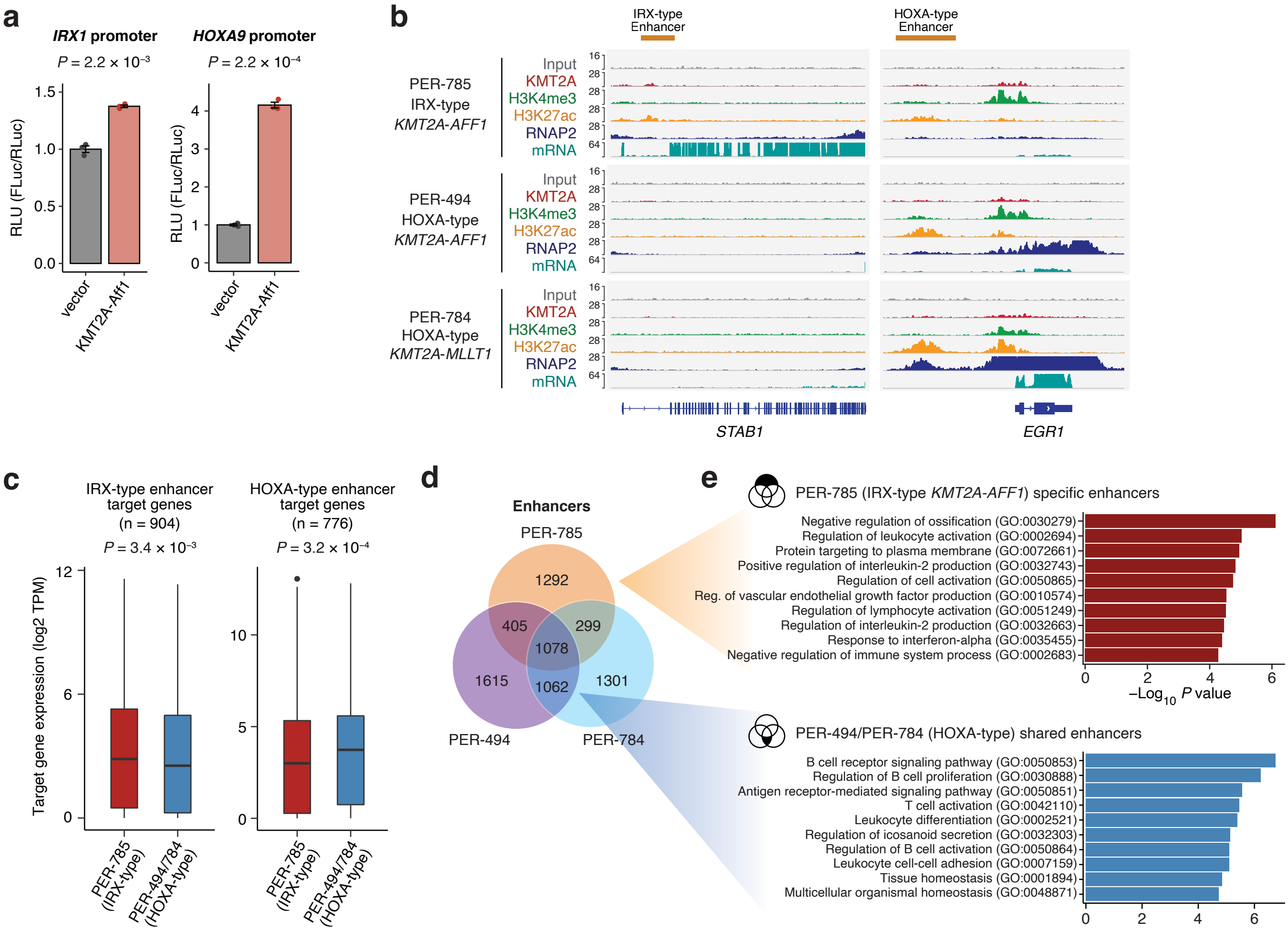

Supplementary Figure 18 | Cell type-specific enhancers defined by differential ChIP signals.

a, Activity of *IRX1* and *HOXA9* promoters in a luciferase assay. Bar plots indicate mean ± standard error of the mean (SEM). Luciferase assays were performed in triplicate (n = 3). Promoter activity was inferred as the ratio of firefly Luciferase (FLuc) to Renilla Luciferase (RLuc). *P* values are from two-sided Welch's t-test. RLU, relative luminescence units. Source data are provided as a Source Data file. **b**, ChIP signals of KMT2A, H3K4me3, H3K27ac and RNAP2 at the *STAB1* and *EGR1* loci are depicted as anexample of IRX-type and HOXA-type enhancers, respectively. **c**, Expression levels of IRX-type and HOXA-type enhancer target genes (n = 904 and 776, respectively) in each type of cell lines. Box plots show median and first/third quartiles. The whisker extends from the smallest to the largest values within 1.5 × IQR from the box hinges. *P* values are from two-sided Wilcoxon rank sum test. **d**, Venn diagram of the number of enhancer calls in each cell line. **e**, Enriched Gene Ontology terms in PER-785-specific enhancers (IRX-type) and common enhancers of PER-494 and PER-784 (HOXA-type).

Supplementary Figure 19

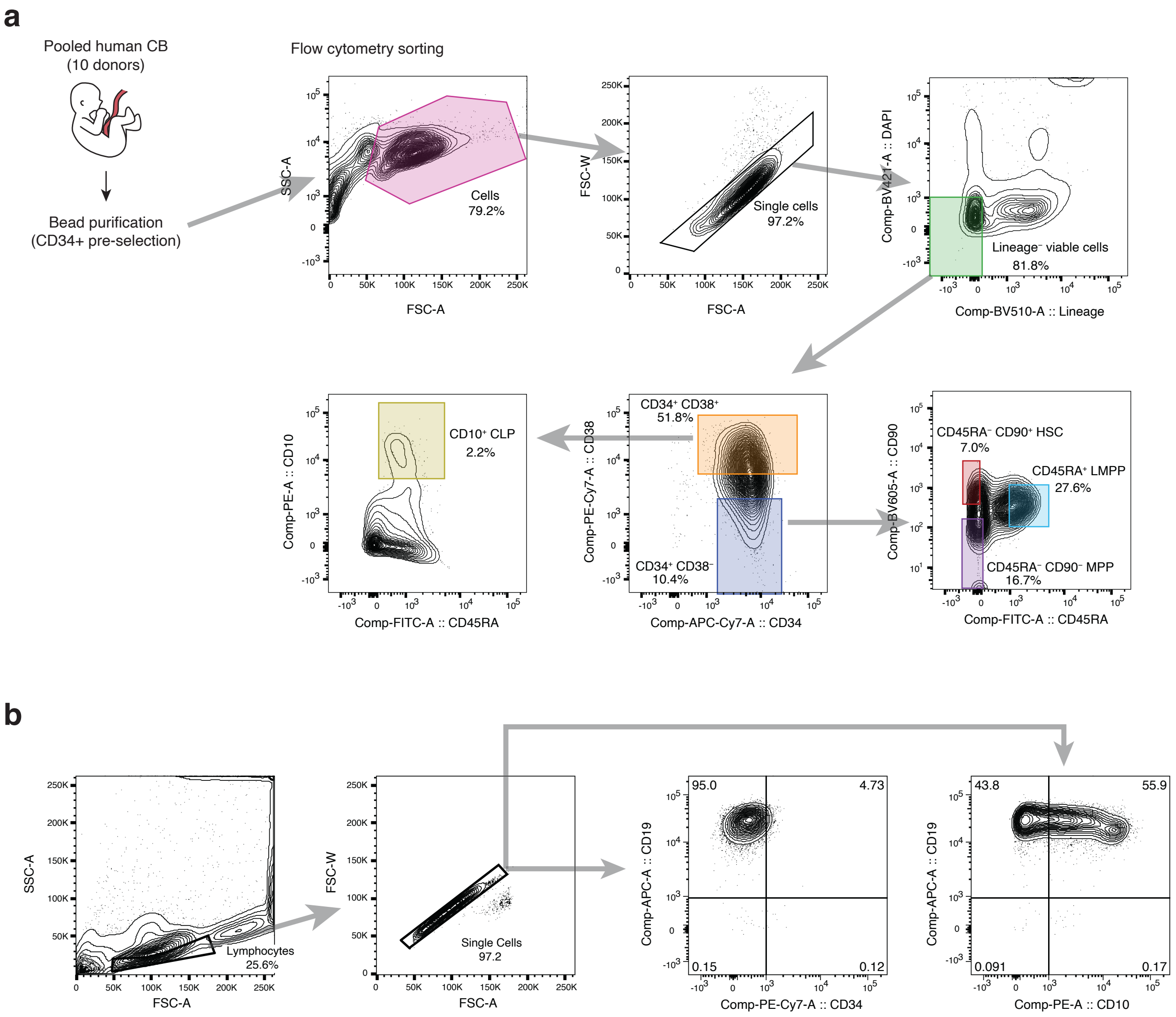

Supplementary Figure 19 I Flow cytometry sorting scheme of human CB HSPCs.

a, Gating strategy for human CB-derived CD34⁺ enriched HSPCs to sort HSCs (Lin⁻ CD34⁺ CD38⁻ CD45RA⁻ CD90⁺), MPPs (Lin⁻ CD34⁺ CD38⁻ CD45RA⁻ CD90⁻), LMPPs (Lin⁻ CD34⁺ CD38⁻ CD45RA⁺) and CLPs (Lin⁻ CD34⁺ CD38⁺ CD10⁺). **b**, Gating strategy for KMT2A-Aff1-transformed HSPCs. Representative gating results for LMPPs (Fig. 7b) are shown.

Supplementary Table 1

	IC1 (n = 14)	IC2 (n = 14)	IC3 (n = 12)	IC4 (n = 9)	IC5 (n = 12)	*P* value[#]
**Age (months)**						0.24
0-3	6 (43%)	9 (64%)	3 (25%)	5 (56%)	3 (25%)	
3-6	6 (43%)	3 (21%)	7 (58%)	1 (11%)	7 (58%)	
6-12	2 (14%)	2 (14%)	2 (17%)	3 (33%)	2 (17%)	
**Sex**						0.27
Female	9 (64%)	9 (64%)	6 (50%)	2 (22%)	5 (42%)	
Male	5 (36%)	5 (36%)	6 (50%)	7 (78%)	7 (58%)	
**CNS involvement**						0.87
Negative	11 (79%)	9 (64%)	8 (67%)	6 (67%)	10 (83%)	
Positive	3 (21%)	4 (29%)	4 (33%)	3 (33%)	2 (17%)	
Not known	0	1 (7%)	0	0	0	
**WBC count (× 10^9/L)**						0.30
<100	5 (36%)	0	1 (8%)	2 (22%)	3 (25%)	
100-300	6 (43%)	9 (64%)	5 (42%)	3 (33%)	5 (42%)	
≥300	3 (21%)	4 (29%)	6 (50%)	4 (44%)	4 (33%)	
Not known	0	1 (7%)	0	0	0	
**Day 8 prednisolone response**						0.51
Good response	5 (36%)	4 (29%)	5 (42%)	4 (44%)	3 (25%)	
Poor response	4 (29%)	0	2 (17%)	2 (22%)	0	
Not known	5 (36%)	10 (71%)	5 (42%)	3 (33%)	9 (75%)	

Supplementary Table 1. Comparison of clinical characteristics in the five ICs.
[#] *P* values are raw *P* values from Fisher's exact test, excluding patients with unknown status.

Supplementary Table 2

IRX1 regulons	*HOXA9* regulons
BMP3	*APOBEC3B*
CALD1	*CBX1*
CYB5R2	*CMPK2*
EFEMP1	*DDX60*
ENAH	*DOCK10*
FILIP1L	*GABARAPL2*
FLT1	*HOXA10*
H19	*HOXA10-AS*
HOTS	*HOXA10-HOXA9*
IGF2BP1	*HOXA4*
LOC283299	*MIR196B*
LRRC32	*NEMP2*
MIR675	*OSTF1*
OR5E1P	*SPINK2*
OVCH2	*VAT1L*
SHANK3	
SLC26A10	

Supplementary Table 2. Positive regulons of *IRX1* and *HOXA9* inferred by RTN.

Supplementary Table 3

Sample ID	Chr	Start	End	Estimated CN	Platform
MLL_10_007	22	40796665	50664259	1.65	WES
MLL_10_007	X	1314871	154444749	2.23	WES
MLL_10_009	11	118356774	118443101	1.03-1.11	WES/Target
MLL_10_011	11	118356774	118915760	1.05-1.20	WES/Target
MLL_10_016	9	21239546	22447000	1.45	WES
MLL_10_026	11	118356774	118528951	1.20-1.34	WES/Target
MLL_10_038	1	220324602	244572850	1.76	WES
MLL_10_038	9	20715258	37440497	1.03	WES
MLL_10_040	9	21239546	33255009	1.08	WES
MLL_10_040	9	36036947	37126253	1.15	WES
MLL_10_040	11	9161226	9492826	1.14	WES
MLL_10_040	11	118065012	118235760	1.04	WES
MLL_10_040	11	118356774	118367946	1.2	Target
MLL_96_011	19	2663758	2833485	1.03	Target
MLL_96_025	16	86251433	87006891	1.03	Target
MLL_96_029	4	2837672	84706373	2.67	Target
MLL_96_029	11	118356774	134021996	2.7	Target
MLL_96_040	6	1335210	169621045	2.23	Target
MLL_96_045	9	36966513	36985922	1.21	Target
MLL_96_045	12	1015540	30851998	1.28	Target
MLL_96_045	13	38290185	114323906	1.3	Target
MLL_96_050	9	21968134	21991832	0.08	Target
MLL_96_050	9	21994115	24741113	1.08	Target
MLL_96_073	11	118356774	119377183	1.23	Target
scmc01	4	2837672	27066105	1.06	Target
scmc01	11	118353415	134021996	1.17	Target
scmc09	9	36825257	37033915	1.04	Target
UT_INF_004	4	2837672	84706373	2.73	Target
UT_INF_004	11	118357518	134021996	2.83	Target

Supplementary Table 3. Copy number alterations in the discovery cohort of 61 infants identified by WES and/or targeted deep sequencing.

Supplementary Table 4

Sample ID	Time point	Gene	Mutation Type	Base/Amino Acid Change	Chr	Start	End	Ref	Obs	VAF[#]
UT_INF_001	Diagnostic	*PTPN11*	missense	NM_002834:exon13:c.G1508C:p.G503A	12	112926888	112926888	G	C	0.27
UT_INF_001	Diagnostic	*FLT3*	missense	NM_004119:exon20:c.A2525G:p.Y842C	13	28592620	28592620	T	C	0.24
UT_INF_001	Diagnostic	*TP53*	frameshift indel	NM_000546:exon7:c.686_687del:p.C229fs	17	7577594	7577595	AC	-	0.24
UT_INF_001_R	Relapse	*PTPN11*	missense	NM_002834:exon13:c.G1508C:p.G503A	12	112926888	112926888	G	C	0.34
UT_INF_001_R	Relapse	*FLT3*	missense	NM_004119:exon20:c.A2525G:p.Y842C	13	28592620	28592620	T	C	0.30
UT_INF_001_R	Relapse	*TP53*	frameshift indel	NM_000546:exon7:c.686_687del:p.C229fs	17	7577594	7577595	AC	-	0.40

Supplementary Table 4. Mutational analysis of the paired diagnostic and relapse samples of UT_INF_001.
[#]VAFs based on amplicon deeq sequencing are shown.

Supplementary Table 5

Sample ID	Chr	Start	End	Estimated CN	Platform
MLL_03_026	2	159389751	161132143	1.1	WES
MLL_03_026	22	22842116	22890417	1.06	WES
MLL_03_027	9	21978959	22005965	1.14	Target
MLL_10_002	X	1314871	154444749	2.49	WES
MLL_10_010	4	123091781	123663024	1.03	WES
MLL_10_031	11	118354186	118367946	1.14	Target

Supplementary Table 5. Copy number alterations identified by WES and/or targeted deep sequencing in 12 infants from the extended cohort.
