# Supplementary material for: Multi-omics analysis defines highly refractory RAS burdened immature subgroup of infant acute lymphoblastic leukemia
Source: Nat Commun. 2022 Aug 30;13:4501. doi: 10.1038/s41467-022-32266-4 (PMC9427775; doi:10.1038/s41467-022-32266-4)
Supplement: Supplementary file 3 — Description of Additional Supplementary Files [file 41467_2022_32266_MOESM3_ESM.pdf]

Description of Additional Supplementary Files

File Name: Supplementary Data 1
Description: Summary of clinicopathological characteristics and experiments for the entire cohort of 84 infants with KMT2A-r leukemia.

File Name: Supplementary Data 2
Description: Quality metrics for RNA sequencing of 61 infants with KMT2A-r leukemia.

File Name: Supplementary Data 3
Description: Fusion transcripts identified in the discovery cohort of 61 infants.

File Name: Supplementary Data 4
Description: Differentially expressed genes for each IC of infant leukemia.

File Name: Supplementary Data 5
Description: Dual-omics molecular markers of infant ALL ICs.

File Name: Supplementary Data 6
Description: Lineage cell type markers obtained from the fetal liver and embryonic single cell transcriptomics studies.

File Name: Supplementary Data 7
Description: List of genes and regions covered for targeted deep sequencing.

File Name: Supplementary Data 8
Description: Validated non-silent mutations detected by WES in 19 infants from the discovery cohort.

File Name: Supplementary Data 9
Description: Non-silent mutations detected by targeted deep sequencing in the discovery cohort of 61 infants.

File Name: Supplementary Data 10
Description: Structural variations in the discovery cohort of 61 infants identified by WES and/or targeted deep sequencing.

File Name: Supplementary Data 11
Description: Validated non-silent mutations detected by WES in 12 infants from the extended cohort.

File Name: Supplementary Data 12
Description: Non-silent mutations detected by targeted deep sequencing in 11 infants from the extended cohort.

File Name: Supplementary Data 13
Description: Structural variations identified by WES and/or targeted deep sequencing in 12 infants from the extended cohort.
