# Supplementary material for: Multi-omics analysis defines highly refractory RAS burdened immature subgroup of infant acute lymphoblastic leukemia
Source: Nat Commun. 2022 Aug 30;13:4501. doi: 10.1038/s41467-022-32266-4 (PMC9427775; doi:10.1038/s41467-022-32266-4)
Supplement: Supplementary file 17 — Reporting Summary [file 41467_2022_32266_MOESM17_ESM.pdf]

nature portfolio

Corresponding author(s): Masatoshi Takagi and Junko Takita

Last updated by author(s): Jul 14, 2022

Reporting Summary

Nature Portfolio wishes to improve the reproducibility of the work that we publish. This form provides structure for consistency and transparency in reporting. For further information on Nature Portfolio policies, see our Editorial Policies and the Editorial Policy Checklist.

Statistics

For all statistical analyses, confirm that the following items are present in the figure legend, table legend, main text, or Methods section.

n/a	Confirmed	
☐	☒	The exact sample size (*n*) for each experimental group/condition, given as a discrete number and unit of measurement
☐	☒	A statement on whether measurements were taken from distinct samples or whether the same sample was measured repeatedly
☐	☒	The statistical test(s) used AND whether they are one- or two-sided<br>*Only common tests should be described solely by name; describe more complex techniques in the Methods section.*
☐	☒	A description of all covariates tested
☐	☒	A description of any assumptions or corrections, such as tests of normality and adjustment for multiple comparisons
☐	☒	A full description of the statistical parameters including central tendency (e.g. means) or other basic estimates (e.g. regression coefficient) AND variation (e.g. standard deviation) or associated estimates of uncertainty (e.g. confidence intervals)
☐	☒	For null hypothesis testing, the test statistic (e.g. *F*, *t*, *r*) with confidence intervals, effect sizes, degrees of freedom and *P* value noted<br>*Give P values as exact values whenever suitable.*
☒	☐	For Bayesian analysis, information on the choice of priors and Markov chain Monte Carlo settings
☒	☐	For hierarchical and complex designs, identification of the appropriate level for tests and full reporting of outcomes
☒	☐	Estimates of effect sizes (e.g. Cohen's *d*, Pearson's *r*), indicating how they were calculated

Our web collection on statistics for biologists contains articles on many of the points above.

Software and code

Policy information about availability of computer code

Data collection	Flow cytometry: BD FACSDiva (9.0 or 8.0.3) software
Data analysis	Data analysis was performed in R (3.6.3, 4.0.2 or 4.0.3) or Python (2.7.10, 3.6.13 or 3.8.6). Softwares/packages used include:<br>(For RNA-seq data analysis) Genomon (2.6.3); STAR (2.5.2a); kallisto (0.44.0); pizzly (0.37.3); DESeq2 (1.26.0); RTN (2.10.1); GSEA (4.1.0); GSVA (1.34.0)<br>(For methylation array analysis) RnBeads (2.4.0); limma (3.42.2); LOLA (1.16.0)<br>(For integrative clustering) SNFtool (2.3.0); CancerSubtypes (1.12.1); ConsensusClusterPlus (1.50.0); GenePattern KNN (version 4); GenePattern KNNXValidation (version 6)<br>(For WES/deep-seq analysis) Genomon (2.6.3); BWA (0.7.8); EBFilter (0.2.1; https://github.com/Genomon-Project/EBFilter); CNACS (0.2.0; https://github.com/papaemmelab/toil_cnacs)<br>(For single-cell RNA-seq analysis) BD Rhapsody Analysis Pipeline (1.9.1); Seurat (4.0.0 or 4.0.1); DoubletFinder (2.0.3); Scanpy (1.8.1); scikit-learn (0.23.2); biomaRt (2.42.1); GSVA (1.38.2); metascape (https://metascape.org/)<br>(For ChIP-seq analysis) Bowtie2 (2.4.2); HOMER (4.11); deeptools (3.5.1); wiggletools (1.2.10); ChIP-Enrich (http://chip-enrich.med.umich.edu)<br>(For survival analysis) survival (3.2-10)<br>(For visualization) pheatmap (1.0.12); ggplot2 (3.3.3); IGV (2.3.97)<br><br>Flow cytometry data were analyzed using FlowJo (10.6.1)

For manuscripts utilizing custom algorithms or software that are central to the research but not yet described in published literature, software must be made available to editors and reviewers. We strongly encourage code deposition in a community repository (e.g. GitHub). See the Nature Portfolio guidelines for submitting code & software for further information.

Data

Policy information about availability of data

All manuscripts must include a data availability statement. This statement should provide the following information, where applicable:

- Accession codes, unique identifiers, or web links for publicly available datasets
- A description of any restrictions on data availability
- For clinical datasets or third party data, please ensure that the statement adheres to our policy

All RNA sequencing, methylation array, whole-exome sequencing, targeted deep sequencing, ChIP sequencing, and single-cell RNA sequencing data generated in this study have been deposited in the Japanese Genotype-phenotype Archive (JGA) under accession code JGAS000385 [https://humandbs.biosciencedbc.jp/en/hum0315-v2]. Access can be requested through the National Bioscience Database Center (NBDC) application system as detailed in the instructions [https://humandbs.biosciencedbc.jp/en/data-use]. Data users must comply with the NBDC guidelines [https://humandbs.biosciencedbc.jp/en/guidelines]. Access is granted for the entire period specified in the users' applications. NBDC administrative staff will respond to requests within one week. Publicly available RNA sequencing data of KMT2A-r infant B-ALL, RNA sequencing data of normal B-cell progenitors and methylation array data of normal B-cell progenitors were downloaded from EGAS00001000246 [https://ega-archive.org/studies/EGAS00001000246], GSE122982 [https://www.ncbi.nlm.nih.gov/geo/query/acc.cgi?acc=GSE122982] and GSE45459 [https://www.ncbi.nlm.nih.gov/geo/query/acc.cgi?acc=GSE45459], respectively. Single-cell RNA sequencing data of fetal liver cells were downloaded from the Development Cell Atlas Web Portal [https://developmentcellatlas.ncl.ac.uk/datasets/hca_liver/data_share/]. The following databases were used as described in Methods: NCBI dbSNP [https://www.ncbi.nlm.nih.gov/snp/], Human Genetic Variation Database [https://www.hgvd.genome.med.kyoto-u.ac.jp] and Ensembl Biomart [https://feb2014.archive.ensembl.org/biomart]. The remaining data are available within the Article, Supplementary Information or Source Data file. Source data are provided with this paper.

Field-specific reporting

Please select the one below that is the best fit for your research. If you are not sure, read the appropriate sections before making your selection.

☒ Life sciences ☐ Behavioural & social sciences ☐ Ecological, evolutionary & environmental sciences

For a reference copy of the document with all sections, see nature.com/documents/nr-reporting-summary-flat.pdf

Life sciences study design

All studies must disclose on these points even when the disclosure is negative.

Sample size	No prior sample size calculations were performed. Considering the rarity of the disease, cases were included on the basis of sample availability.
Data exclusions	In single-cell RNA sequencing analysis, low quality single cells were excluded, as described in the Methods.
Replication	Similar patient molecular profiles were confirmed using a discovery cohort (n = 61) and an independent extended cohort (n = 23). ChIP-seq was performed in duplicate with independent cell cultures. IP and sequencing library preparation for the duplicate were performed independently at different points in time. RNA sequencing of infant ALL cell lines were performed in triplicate with independent cell cultures. Luciferase assays were performed in triplicate. CB transformation was performed using pooled CB HSPCs from different individuals (n=10) to minimize batch effects and was independently repeated three times.
Randomization	Patient randomization is not relevant for this study. All patient samples were collected at diagnosis of infant ALL and underwent no prior treatment/randomization. 293T cells and MS-5 stroma cells were randomly allocated to experimental batches.
Blinding	Analyses with patient characteristics as a variable (e.g., survival analysis) were not performed until data-driven unsupervised clustering results were assigned. Investigators were not blind to experimental groups as knowledge of groups and cell type identities was necessary to perform the experiments and analyses.

Reporting for specific materials, systems and methods

We require information from authors about some types of materials, experimental systems and methods used in many studies. Here, indicate whether each material, system or method listed is relevant to your study. If you are not sure if a list item applies to your research, read the appropriate section before selecting a response.

Materials & experimental systems

n/a	Involved in the study
☐	☒ Antibodies
☐	☒ Eukaryotic cell lines
☒	☐ Palaeontology and archaeology
☒	☐ Animals and other organisms
☐	☒ Human research participants
☒	☐ Clinical data
☒	☐ Dual use research of concern

Methods

n/a	Involved in the study
☐	☒ ChIP-seq
☐	☒ Flow cytometry
☒	☐ MRI-based neuroimaging

Antibodies

Antibodies used

Antibody details are described in the Methods and are listed below:

(For flow cytometry analysis)

CD34-APCCy7 (BioLegend, 343513, 1:500)
CD38-PECy7 (BioLegend, 303515, 1:500)
CD90-biotin (BioLegend, 328106, 1:500)
CD45RA-FITC (BioLegend, 304105, 1:100)
CD10-PE (BioLegend, 312204, 1:100 for sorting and 1:500 for analysis)
Streptavidin-BV605 (BioLegend, 405229, 1:500)
human hematopoietic lineage cocktail BV510 (BioLegend, 348807, 1:100)
CD34-PECy7 (BioLegend, 343616, 1:500)
CD19-APC (BioLegend, 302212, 1:500)
CD19-APC (Miltenyi Biotec, 130-091-248, 1:33)
CD45 VioBlue (Miltenyi Biotec, 130-113-122, 1:33)
DAPI (BioLegend, 422801)
7-AAD (Miltenyi Biotec, 130-111-568)

(For ChIP-seq)

KMT2A (N-terminal, Cell Signaling, 14689, 1:400)
H3K4me3 (Active Motif, 39159, 1:400)
H3K27ac (MABI, 308-34843, 1:400)
RNAP2 (Millipore, 05-623, 1:400)

Validation

All antibodies are commercially available and were validated by manufactures. The validation of each primary antibodies for the reactive species and applications are described below.

CD34-APCCy7 (BioLegend, 343513) - Reactive species: Human (Cross-Reactivity: Cynomolgus), Application: Flow cytometry
CD38-PECy7 (BioLegend, 303515) - Reactive species: Human, Chimpanzee, Horse, Cow, Application: Flow cytometry
CD90-biotin (BioLegend, 328106) - Reactive species: Human, African Green, Baboon, Cynomolgus, Pigtailed Macaque, Rhesus, Swine (Pig, Porcine), Application: Flow cytometry
CD45RA-FITC (BioLegend, 304105) - Reactive species: Human (Cross-Reactivity: Chimpanzee), Application: Flow cytometry
CD10-PE (BioLegend, 312204) - Reactive species: Human, African Green, Baboon, Capuchin monkey, Chimpanzee, Cynomolgus, Rhesus, Application: Flow cytometry
Streptavidin-BV605 (BioLegend, 405229) - Human, Mouse, Rat, All Species, Application: Flow cytometry
human hematopoietic lineage cocktail BV510 (BioLegend, 348807) - Reactive species: Human, Application: Flow cytometry
CD34-PECy7 (BioLegend, 343616) - Reactive species: Human (Cross-Reactivity: Cynomolgus, Rhesus), Application: Flow cytometry
CD19-APC (BioLegend, 302212) - Reactive species: Human, Chimpanzee, Rhesus, Application: Flow cytometry
CD19-APC (Miltenyi Biotec, 130-091-248) - Reactive species: Human, Application: Flow cytometry
CD45 VioBlue (Miltenyi Biotec, 130-113-122) - Reactive species: Human, Application: Flow cytometry

KMT2A (N-terminal, Cell Signaling, 14689) - Reactive species: Human, Mouse, Rat, Monkey, Application: ChIP
H3K4me3 (Active Motif, 39159) - Reactive species: Budding Yeast, Human, Mouse, Wide Range Predicted, Application: ChIP
H3K27ac (MABI, 308-34843) - Reactive species: Human, Application: ChIP
RNAP2 (Millipore, 05-623) - Reactive species: Human, Rat, Mouse, Yeast (S. cerevisiae), Application: ChIP

Eukaryotic cell lines

Policy information about cell lines

Cell line source(s)

Infant ALL cell lines (PER-494, PER-784, PER-785) were established and provided by Dr. Rishi S Kotecha and Dr. Mark N Cruickshank at University of Western Australia. MS-5 cells were established and provided by Dr. Kazuhiro J Mori at Niigata

	University. HEK293T cells were purchased from ATCC (Cat #CRL-11268)
Authentication	All cell lines used in this study were authenticated by short-tandem repeat analyses.
Mycoplasma contamination	All cell lines tested negative for mycoplasma contamination.
Commonly misidentified lines (See ICLAC register)	No commonly misidentified cell lines were used in this study.

Human research participants

Policy information about studies involving human research participants

Population characteristics	Diagnostic bone marrow or peripheral blood samples from 84 infants (aged <12 months) with B-ALL (n = 82) or B/M MPAL (n = 2) were procured from the Japan Children's Cancer Group (JCCG) and its affiliated hospitals. Further details on patient characteristics are provided in Supplementary Data 1.
Recruitment	Patients were recruited through the JCCG/JPLSG and its affiliated hospitals and were treated according to MLL-10 (n = 33), MLL03 (n = 12), MLL96/98 (n = 37) or other protocols (n = 2). Considering the rarity of the disease, cases were included on the basis of sample availability.
Ethics oversight	Written informed consent was obtained from the parents and/or legal guardians in accordance with the Declaration of Helsinki. The research protocol was approved by the Human Genome, Gene Analysis Research Ethics Committee of the University of Tokyo (G0948-(19)), the Ethics Committee of Kyoto University Graduate School and Faculty of Medicine (G-1030-8), the Review Board of Tokyo Medical and Dental University (G2000-193 and G2000-103) and the Review Board of Japan Pediatric Leukemia Study Group (JPLSG) (041).

Note that full information on the approval of the study protocol must also be provided in the manuscript.

ChIP-seq

Data deposition

- [x] Confirm that both raw and final processed data have been deposited in a public database such as GEO.
- [x] Confirm that you have deposited or provided access to graph files (e.g. BED files) for the called peaks.

Data access links *May remain private before publication.*	JGAS000385
Files in database submission	PER494_H3K27ac_rep1.bam<br>PER494_H3K27ac_rep2.bam<br>PER494_H3K4me3_rep1.bam<br>PER494_H3K4me3_rep2.bam<br>PER494_Input_rep1.bam<br>PER494_Input_rep2.bam<br>PER494_MLLN_rep1.bam<br>PER494_MLLN_rep2.bam<br>PER494_RNAP2_rep1.bam<br>PER494_RNAP2_rep2.bam<br>PER784_H3K27ac_rep1.bam<br>PER784_H3K27ac_rep2.bam<br>PER784_H3K4me3_rep1.bam<br>PER784_H3K4me3_rep2.bam<br>PER784_Input_rep1.bam<br>PER784_Input_rep2.bam<br>PER784_MLLN_rep1.bam<br>PER784_MLLN_rep2.bam<br>PER784_RNAP2_rep1.bam<br>PER784_RNAP2_rep2.bam<br>PER785_H3K27ac_rep1.bam<br>PER785_H3K27ac_rep2.bam<br>PER785_H3K4me3_rep1.bam<br>PER785_H3K4me3_rep2.bam<br>PER785_Input_rep1.bam<br>PER785_Input_rep2.bam<br>PER785_MLLN_rep1.bam<br>PER785_MLLN_rep2.bam<br>PER785_RNAP2_rep1.bam<br>PER785_RNAP2_rep2.bam<br>PER494_H3K27ac_rep1.bw<br>PER494_H3K27ac_rep2.bw

PER494_H3K4me3_rep1.bw
PER494_H3K4me3_rep2.bw
PER494_Input_rep1.bw
PER494_Input_rep2.bw
PER494_MLLN_rep1.bw
PER494_MLLN_rep2.bw
PER494_RNAP2_rep1.bw
PER494_RNAP2_rep2.bw
PER784_H3K27ac_rep1.bw
PER784_H3K27ac_rep2.bw
PER784_H3K4me3_rep1.bw
PER784_H3K4me3_rep2.bw
PER784_Input_rep1.bw
PER784_Input_rep2.bw
PER784_MLLN_rep1.bw
PER784_MLLN_rep2.bw
PER784_RNAP2_rep1.bw
PER784_RNAP2_rep2.bw
PER785_H3K27ac_rep1.bw
PER785_H3K27ac_rep2.bw
PER785_H3K4me3_rep1.bw
PER785_H3K4me3_rep2.bw
PER785_Input_rep1.bw
PER785_Input_rep2.bw
PER785_MLLN_rep1.bw
PER785_MLLN_rep2.bw
PER785_RNAP2_rep1.bw
PER785_RNAP2_rep2.bw

Genome browser session (e.g. UCSC): NA

Methodology

Replicates: ChIP-seq was performed in duplicate with independent cell cultures. IP and sequencing library preparation were performed at different points in time.

Sequencing depth: Libraries of the precipitated DNA were sequenced on an Illumina HiSeq 2500 Platform using 50 bp single-end mode. On average, the total number of reads was 31.8 million per sample, of which 69.7% were uniquely mapped.

Antibodies:
KMT2A (N-terminal, Cell Signaling, 14689)
H3K4me3 (Active Motif, 39159)
H3K27ac (MABI, 308-34843)
RNAP2 (Millipore, 05-263)

Peak calling parameters: Sequencing data were mapped to hg19 using Bowtie2 with the default parameters. Cell line-specific enhancers were called using HOMER with the following command: getDifferentialPeaksReplicates.pl -t <H3K27ac rep1> <H3K27ac rep2> -b <H3K4me3 rep1> <H3K4me3 rep2> -i <Input rep1> <Input rep2> -genome hg19 -style histone

Data quality: Only reads with mapping quality score ≥ 20 and aligned to autosomal and sex chromosomes were used for downstream analyses.

Software: Bowtie2 (2.4.2); HOMER (4.11); deeptools (3.5.1); wiggletools (1.2.10); ChIP-Enrich (http://chip-enrich.med.umich.edu)

Flow Cytometry

Plots

Confirm that:

- [x] The axis labels state the marker and fluorochrome used (e.g. CD4-FITC).
- [x] The axis scales are clearly visible. Include numbers along axes only for bottom left plot of group (a 'group' is an analysis of identical markers).
- [x] All plots are contour plots with outliers or pseudocolor plots.
- [x] A numerical value for number of cells or percentage (with statistics) is provided.

Methodology

Sample preparation: Cells were stained by fluoro-conjugated antibodies for 30 min at 4˚ C. After staining, cells were washed with cold PBS for several times, and were resuspended with PBS containing 2% FBS.

Instrument	Cells were analysed on a FACSCanto II, a FACSAria III or a FACSAria Fusion (BD Biosciences).
Software	Data were collected using BD FACSDiva software and were analyzed with FlowJo software.
Cell population abundance	Cell populations were sorted at >95% purity post-sort in pilot experiments, as determined by flow cytometry.
Gating strategy	Cells were gated for size exclusion (FSC-A/SSC-A) followed by doublet exclusion (FSC-A/FSC-W). The following gating strategies are shown in Supplementary Figures 6 and 8. Boundaries between negative and positive were determined by single stained control.

☒ Tick this box to confirm that a figure exemplifying the gating strategy is provided in the Supplementary Information.
